# Unprecedented Monoterpenoid Polyprenylated Acylphloroglucinols with a Rare 6/6/5/4 Tetracyclic Core, Enhanced MCF-7 Cells’ Sensitivity to Camptothecin by Inhibiting the DNA Damage Response

**DOI:** 10.3390/biomedicines9101473

**Published:** 2021-10-14

**Authors:** Xiang-Zhong Liu, Mi Zhou, Chun-Chun Du, Hong-Hong Zhu, Xi Lu, Shou-Lun He, Guang-Hui Wang, Ting Lin, Wen-Jing Tian, Hai-Feng Chen

**Affiliations:** Fujian Provincial Key Laboratory of Innovative Drug Target, School of Pharmaceutical Sciences, Xiamen University, Xiamen 361102, China; lxz_0501@126.com (X.-Z.L.); zmxmuer@163.com (M.Z.); DuChunchun97@163.com (C.-C.D.); 17862809369@163.com (H.-H.Z.); cjyzwdwrm@stu.xmu.edu.cn (X.L.); hesl0330@163.com (S.-L.H.); guanghui@xmu.edu.cn (G.-H.W.); lintin@xmu.edu.cn (T.L.)

**Keywords:** Guttiferae, *Hypericum elodeoides*, acylphloroglucinol, biosynthetic pathway, DNA damage response inhibitor

## Abstract

(±)-Hypersines A–C (**1**–**3**), the three pairs of enantiomerically pure monoterpenoid polyprenylated acylphloroglucinols with an unprecedented 6/6/5/4 fused ring system, were isolated from *Hypericum elodeoides*. Their structures, including absolute configurations, were elucidated by comprehensive spectroscopic data, single-crystal X-ray diffraction, and quantum chemical calculations. The plausible, biosynthetic pathway of **1**–**3** was proposed. Moreover, the bioactivity evaluation indicated that **1a** might be a novel DNA damage response inhibitor, and could enhance MCF-7 cell sensitivity to the anticancer agent, camptothecin.

## 1. Introduction

Polycyclic polyprenylated acylphloroglucinols (PPAPs) have been a research hotspot among the chemical community due to their fascinating structures and diversiform pharmacological activities [1]. Hitherto, more than 800 PPAPs have been reported as isolated from the plants of the Guttiferae family, especially the genera *Hypericum* and *Garcinia* [2,3,4]. Monoterpenoid polyprenylated acylphloroglucinols (MTPAPs), a special type of structurally diverse PPAPs, are generally decorated with a geranyl or a cyclic monoterpene fragment at C-3 of the phloroglucinol ring [5,6]. To date, approximately 110 MTPAPs (Table S1) have been identified, and these can be divided into several types due to their different ring systems, including the uncyclized MTPAPs [7,8] and cyclized MTPAPs with 6/5, 6/6, 6/5/6, 6/6/6, and 6/7/5 ring systems (Figure S1) [6,9,10,11]. The MTPAPs exhibit a broad range of biological activities including cytotoxicity, anti-HIV, anti-inflammatory, anthelmintic, and antibacterial activities [12,13,14,15,16,17]. The intriguing structures, as well as the potential bioactivities of MTPAPs, have garnered our attention.

**Figure 1:**
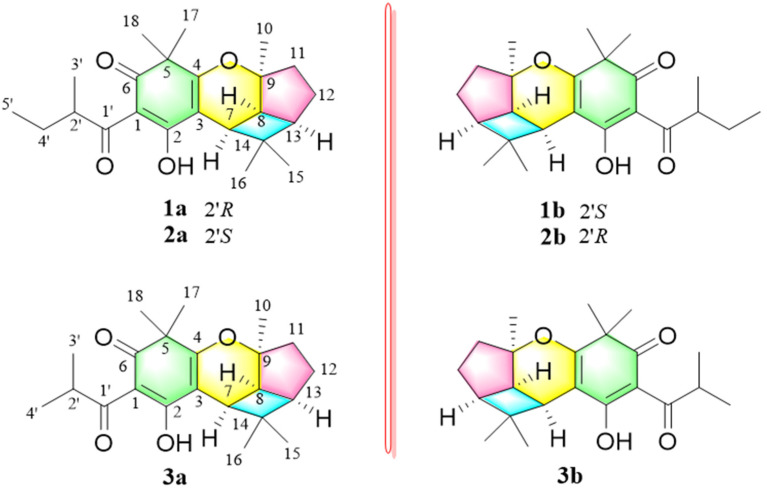
Structures of compounds **1**–**3**.

*Hypericum elodeoides*, a medicinal herb belonging to the genera *Hypericum*, is traditionally used in China for the treatment of stomatitis, infantile pneumonia, and mastitis. Previous phytochemical investigations of *H. elodeoides* have led to several PPAPs derivatives with various skeletons, xanthones, and flavonoids [6,18,19,20,21,22]. In this study, three pairs of novel MTPAPs with unprecedented 6/6/5/4 tetracyclic scaffolds, (±)-Hypersines A–C (**1**–**3**), were isolated and identified from *H. elodeoides* (Figure 1). All featured an unusual, four-membered carbocycle with large tension, concurrently fused with five- and six-membered rings in the monoterpene moiety, these fused with the methylated acylphloroglucinol core and led to a new pattern of ring systems. A four-membered carbocycle existed in several PPAPs, such as hyperjapones H–I [23] and hyperjapones B–E [24], however, the four-membered ring in **1**–**3** possessed a rather different parallel and biosynthetic pathway. Interestingly, compounds **1** and **2** were obtained as C-2’ epimers, and both existed as enantiomers. The epimers and racemic mixtures could be successfully separated by the chiral column. Herein, we report the isolation, structural elucidation, proposed biosynthetic pathway, and biological evaluations of (±)-Hypersines A–C.

## 2. Materials and Methods

### 2.1. General Methods

UV spectra were detected on a Shimadzu UV-2600 UV-visible spectrophotometer (Shimadzu, Tokyo, Japan). IR spectra were recorded on a Bruker ALPHA FT-IR spectrometer (Bruker, Rheinstetten, Germany), with KBr pellets. Optical rotations were measured with a Rudolph Autopol IV/IV-T automatic polarimeter (Rudolph Research Analytical, NJ, USA). Extensive NMR spectra were acquired on Bruker Avance 600 Ⅲ spectrometers (Bruker, Rheinstetten, Germany), using chloroform-d as a solvent. HRESIMS experiments were conducted on a Thermo Scientific Q Exactive Quadrupole–Orbitrap mass spectrometer (Thermo Fisher Scientific Inc., Waltham, MA, USA). ECD spectra were obtained on a MOS-500 circular dichroism spectrometer (BioLogic, Seyssinet-Pariset, France) in methanol. Silica gel (200–300 mesh, Qingdao Marine Chemical Inc., Qingdao, China), MCI gel CHP 20P/P120 (75–150 μm, Mitsubishi Chemical Industries, Tokyo, Japan), and ODS (50 μm, YMC Co. Ltd., Kyoto, Japan) were applied as packing for column chromatography. Semi-preparative HPLC was performed on a Shimadzu LC-16P pump system (Shimadzu, Tokyo, Japan), with a UV detector and a YMC-Pack ODS-A (5 μm, 10 × 250 mm) (YMC Co. Ltd., Kyoto, Japan) or a COSMOSIL 5C_18_-AR-Ⅱ (5 μm, 10 × 250 mm)( Nacalai Tesque, Kyoto, Japan) column. The enantioseparation was carried out by using CHIRALPAK^®^ IG column (5 μm, 10 × 250 mm, Daicel Chiral Technologies Co. Tokyo, Japan). Fractions were monitored by TLC (Qingdao Marine Chemical Inc., Qingdao, China), and visualized under a UV lamp at either 254 nm or 365 nm.

### 2.2. Plant Material

The whole plants of *H. elodeoides* were collected from Yunnan Province, People’s Republic of China, in August 2016. The plant was authenticated by Prof. Zhen-Ji Li (College of the Environment and Ecology, Xiamen University), and a voucher specimen (ID HE201608) deposited at the School of Pharmaceutical Sciences, Xiamen University.

### 2.3. Extraction and Isolation

The air-dried and powered herbs of *H. elodeoides* (7500.0 g) were soaked in methanol (3 × 75 L) at room temperature three times. The crude extract (800.0 g) was subjected to the silica gel column chromatography, eluting with CH_2_Cl_2_, EtOAc, and methanol to afford three fractions (Fr. A–C). Then Fr. A (69.5 g) was divided into six parts (Fr. A1–6) by MCI gel column (MeOH−H_2_O, from 8:2 to 10:0, *v*/*v*). Fr. A4 (2.55 g) was fractionated by silica gel with a petroleum ether—ethyl acetate gradient elution system (100:0 to 10:1, *v*/*v*) to provide six fractions: Fr. A4.1–4.6. Fr. A4.3 (0.6606 g) was further separated by pre-HPLC (ACN–H_2_O, 90:10, *v*/*v*) to obtain Fr. A4.3.1–6. The fraction, Fr. A4.3.6 (0.0148 g), was purified by COSMOSIL 5C_18_-AR-Ⅱ (5 μm, 10 × 250 mm) column (MeOH-H_2_O, 85:15, *v*/*v*) to yield the mixtures of compounds **1** and **2**. Fr. A4.3.5 (0.0392 g) was separated by COSMOSIL 5C_18_-AR-Ⅱ (5 μm, 10 × 250 mm) column (MeOH-H_2_O, 80:20, *v*/*v*) to yield compound **3**. The chiral resolution of **1**, **2,** and **3** were carried out by CHIRALPAK^®^ IG column (MeOH-H_2_O, 80:20 and 90:10, *v*/*v*) to yield **1a** (2.2 mg), **1b** (2.8 mg), **2a** (2.5 mg), **2b** (2.1 mg), **3a** (1.2 mg), and **3b** (1.0 mg).

#### 2.3.1. Hypersine A

UV (CH_3_OH) *λ*_max_ (log *ε*) = 203 (3.87), 226 (3.60), 251 (3.56), 276 (3.46), 332 (3.73) nm; IR *ν*_max_ = 3314, 2955, 1657, 1558, 1460, 1419, 1346, 1192, 1133 cm^−1^; see Table 1 for ^1^H NMR (600 MHz) and ^13^C NMR (150 MHz) data; HRESIMS [M + H]^+^ m/z 373.2375 (calculated for C_23_H_33_O_4_^+^ 373.2373).

**1a**: Light brown oil ${\left[\alpha \right]}_{D}^{28}$ + 71.0 (*c* 0.18, CH_3_OH); ECD (CH_3_OH) *λ* (Δ*ε*) 211 (−12.15), 239 (−2.42), 255 (−4.17), 329 (+5.85) nm.

**1b**: Light brown oil, ${\left[\alpha \right]}_{D}^{28}$ − 67.0 (*c* 0.23, CH_3_OH); ECD (CH_3_OH) *λ* (Δ*ε*) 207 (+7.66), 239 (+0.53), 253 (+1.54), 330 (−4.07) nm.

#### 2.3.2. Hypersine B

UV (CH_3_OH) *λ*_max_ (log *ε*) = 203 (3.91), 226 (3.61), 251 (3.59), 276 (3.49), 332 (3.73) nm; IR *ν*_max_ = 3291, 2963, 1693, 1656, 1615, 1525, 1459, 1415, 1189, 1134 cm^−1^; see Table 1 for ^1^H NMR (600 MHz) and ^13^C NMR (150 MHz) data; HRESIMS [M + H]^+^ m/z 373.2375 (calculated for C_23_H_33_O_4_^+^ 373.2373).

**2a**: Light brown oil, ${\left[\alpha \right]}_{D}^{28}$ + 88.5 (*c* 0.21, CH_3_OH); ECD (CH_3_OH) *λ* (Δ*ε*) 214 (−7.16), 240 (−1.18), 254 (−2.31), 333 (+3.77) nm.

**2b**: Light brown oil, ${\left[\alpha \right]}_{D}^{28}$ − 97.1 (*c* 0.18, CH_3_OH); ECD (CH_3_OH) *λ* (Δ*ε*) 210 (+8.06), 238 (+0.55), 253 (+1.94), 333 (−4.74) nm.

#### 2.3.3. Hypersine C

UV (CH_3_OH) *λ*_max_ (log *ε*) = 203 (3.56), 227 (3.35), 250 (3.33), 278 (3.22), 332 (3.39) nm; IR *ν*_max_ = 2976, 2881, 1653, 1533, 1458, 1372, 1200, 1117 cm^−1^; see Table 1 for ^1^H NMR (600 MHz) and ^13^C NMR (150 MHz) data; HRESIMS [M + H]^+^ m/z 359.2212 (calculated for C_22_H_31_O_4_^+^ 359.2217).

**3a**: Light brown oil, ${\left[\alpha \right]}_{D}^{28}$ + 24.0 (*c* 0.10, CH_3_OH); ECD (CH_3_OH) *λ* (Δ*ε*) 210 (−4.62), 241 (−0.77), 254 (−1.09), 326 (+2.63) nm.

**3b**: Light brown oil, ${\left[\alpha \right]}_{D}^{28}$ − 33.7 (*c* 0.08, CH_3_OH); ECD (CH_3_OH) *λ* (Δ*ε*) 213 (+4.67), 237 (+1.04), 253 (+1.33), 328 (−2.73) nm.

### 2.4. Quantum Chemical Calculation of ^13^C NMR

All structures were optimized at the b3lyp/6-31+g (d, p) level for all conformations using Gaussian09 [25]. The ^13^C NMR shielding constants were computed using the Gauge Independent Atomic Orbital (GIAO) method [26] at the mpw1pw91/6-311+g (2d, p) level, with SMD in chloroform using Gaussian09. The final, calculated ^13^C NMR chemical shifts were obtained by averaging the ^13^C NMR chemical shifts of the optimized conformers according to the Boltzmann distribution theory and their relative Gibbs free energy (∆G).

### 2.5. Quantum Chemical Calculation of ECD Spectra

Monte Carlo conformational searches were carried out by means of the Spartan’s 10 software, using the Merck Molecular Force Field (MMFF). The conformers with a Boltzmann population of over 2% were chosen for ECD calculations, and were then initially optimized at B3LYP/6-311g (2d, p) level in MeOH using the CPCM polarizable conductor calculation model. The theoretical calculation of ECD was conducted in MeOH using time-dependent density-functional theory (TDDFT) at the B3LYP/6-311g (2d, p) level, for all conformers. The rotatory strengths for 60 excited states were calculated. ECD spectra were generated using the SpecDis 1.7.1 program (University of Würzburg, Würzburg, Germany) and GraphPad Prism 5 (University of California San Diego, CA, USA) from dipole-length rotational strengths, and by applying Gaussian band shapes with sigma = 0.3 eV.

### 2.6. Single-Crystal X-ray Diffraction Analysis of ***2b***

Crystal data for **2b**: C_23_H_31_O_4_ (M = 371.48 g/mol), orthorhombic, space group P2_1_2_1_2_1_ (no. 19), a = 9.25724(11) Å, b = 10.10414(12) Å, c = 21.6403(3) Å, V = 2024.16(4) Å3, Z = 4, T = 149.99(10) K, μ(Cu Kα) = 0.653 mm^−^^1^, Dcalc = 1.219 g/cm^3^, 11,032 reflections measured (8.172° ≤ 2Θ ≤ 147.862°), 4009 unique (R_int_ = 0.0246, R_sigma_ = 0.0261), which were used in all calculations. The final R_1_ was 0.0332 (I > 2σ(I)), and wR_2_ was 0.0876 (all data). The goodness of fit for F^2^ was 1.053. Flack/Hooft parameter = 0.01(7)/0.02(7).

### 2.7. Cell Culture

The MCF-7 cell line was obtained from the National Collection of Authenticated cell cultures (Shanghai, China). The cells were cultured in Dulbecco’s Modified Eagle’s Medium (DMEM, BasalMedia, Shanghai, China), containing 10% FBS (Thermo Fisher Scientific, Waltham, MA, USA) at 37 °C, and 5% CO_2_.

### 2.8. MTT Assay

The cell viability was evaluated by the MTT assay. MCF-7 cells were seeded in a 96-well plate, at a density of 1 × 10^4^ cells/well. The cells were grown overnight at 37 °C with humidified 5% CO_2_, and then treated with compounds **1a**–**3b** (20 μM) for 48 h. The supernatants were discarded and 15 μL MTT reagent, as well as 60 μL DMEM, were added. After incubating for 4 h, the supernatants were removed and DMSO was added. The absorbances were measured at 490 nm by the Thermo Multiskan MK3 (Thermo Scientific, Waltham, MA, USA). The cell viability was analyzed as follows:

Growth Rate (%) = [(ODsample-ODblank)/(ODcontrol-ODblank)] × 100%

### 2.9. Western Blotting

Western blotting was conducted as described [27]. After exposure to compounds and CPT for 48 h, cells were lysed by the RIPA buffer with a protease inhibitor and a phosphatase inhibitor (MCE). Cell lysates were separated by sodium dodecyl sulfate polyacrylamide gel electrophoresis (SDS-PAGE) and transferred into the PVDF membrane. The membranes were blocked in no-fat milk with TBST and incubated with primary antibody at 4 °C overnight. Then, the membranes were washed 3 times in TBST and incubated with secondary antibody at room temperature. After being washed, the target proteins on the membranes were detected by chemi-luminescence reagent (Advansta, San Jose, CA, USA).

### 2.10. Cell Cycle Analysis

The MCF-7 cells were seeded in 6 cm dishes. After exposure to CPT with or without compound **1a** for 48 h, the cells were harvested by trypsin with no EDTA, and fixed in cold 75% ethanol overnight. Following centrifuge (1000 rpm, 5 min room temperature) and removing the supernatant, the cells were incubated with 10 μg/mL RNase in PBS at 37 °C for 30 min, and with PI staining buffer at room temperature for 5 min in the dark. The cell cycle distribution was analyzed by flow cytometry (CytoFlex, Beckman Coulter, Los Angeles, CA, USA).

### 2.11. Statistical Analysis

The data were shown as mean ± SD, and the differences between samples were evaluated statistically with ANOVA analysis and Tukey’s posteriori comparison through GraphPad Prism 6.0. Differences were statistically significant at *p* < 0.05.

## 3. Results

### 3.1. Isolation of Compounds ***1**–**3***

The whole plants of *H. elodeoides* were extracted with methanol at room temperature. The crude extract was subjected to the silica gel column chromatography, eluting with CH_2_Cl_2_, EtOAc, and methanol. Compounds **1**–**3** were isolated from the CH_2_Cl_2_ fraction by a series of column chromatography. Then, three pairs of enantiomers, (±)-Hypersines A–C, were purified by chiral HPLC separation with CHIRALPAK^®^ IG column.

### 3.2. Structural Elucidation of Compounds ***1**−**3***

The structures, including absolute configurations of isolated new compounds (±)-hypersines A–C, were elucidated by comprehensive spectroscopic data, single-crystal X-ray diffraction, and quantum chemical calculations.

(±)-Hypersine A (**1a**/**1b**) and (±)-Hypersine B (**2a**/**2b**) were obtained as two pairs of C-2′ epimers. The mixtures of **1a**/**1b** and **2a**/**2b** performed a single chromatographic peak in the conventional RP-18 column, but could be separated into four peaks by chiral columns (Figure S2). The correspondence of the two pairs of enantiomers, **1a**/**1b** and **2a**/**2b**, were ensured by comparing their ^1^H NMR data (Figure S3). The molecular formulae of **1a**/**1b** and **2a**/**2b** were assigned as C_23_H_32_O_4_, on the basis of the HR-ESI-MS molecular ion peak at m/z 373.2375 [M + H]^+^ (calculated for C_23_H_33_O_4_^+^, 373.2379) (**1a**/**1b**) and m/z 373.2374 [M + H]^+^ (calculated for C_23_H_33_O_4_^+^, 373.2379) (**2a**/**2b**), indicating eight indices of hydrogen deficiency. The characteristic absorption bands of UV spectra (203, 226, 251, 276, and 332 nm) and IR spectra (3314 and 1657 cm^−1^) deduced the presence of an enolic 1,3-diketone moiety in **1 [28,29,30]**. The ^1^H NMR and HSQC spectra of **1** showed the signals assignable to five singlet methyl groups (*δ*_H_ 1.40 (s), 1.36 (s), 1.34 (s), 1.32 (s) and 0.80 (s)), one doublet methyl (*δ*_H_ 1.14 (d, *J* = 7.2)), and one triplet methyl (*δ*_H_ 0.90 (t, *J* = 7.2)) The ^13^C NMR and DEPT-135 afforded twenty-three carbon resonances, including seven methyls, three methylenes, four methines, and nine quaternary carbons. The downfield signals at *δ*_C_ 207.9 (C-1’), 197.3 (C-6), 189.4 (C-2), and 105.0 (C-3) suggested the existence of an enol-*β*-triketone moiety [22,25,26]. The above data indicated that **1** was similar to (±)-Hyperjaponol A [30], with a filicinic acid core, and proposed to be a derivative of MTPAPs.

**Figure 2:**
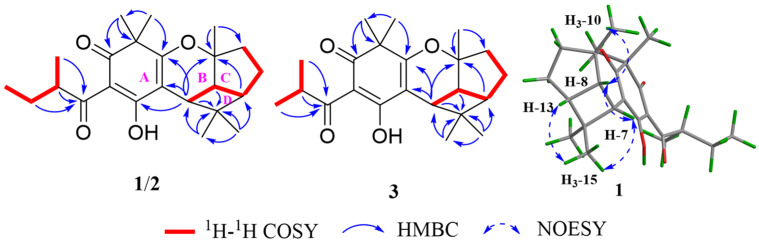
Key 2D NMR correlations of **1**–**3**.

The planning structure of **1** was constructed by analyzing its 2D NMR data (Figure 2). The spin systems of H-7/H-8/H-13/H2-12/H2-11 and H3-2’/H-3’/H2-4’/H3-5’ in the ^1^H-^1^H COSY spectrum indicated the existence of two fragments: CH-7–CH-8–CH-13–CH_2_-12–CH_2_-11 and CH_3_-3’–CH-2’–CH-4’–CH_3_-5’. The HMBC correlations from CH_3_-17 and CH_3_-18 to C-4, C-5, and C-6, and from H-7 to C-3, C-4, and C-9 confirmed the presence of the filicinic acid core (ring A), and that the oxygen heterocycles (ring B) were fused to ring A. By referring to (±)-Hyperjaponol A, the 2-methylbutanoyl group was connected to the C-1 of ring A according to the HMBC correlations from H-2’, H-3’, and H-4’ to C-1’. In addition, the HBMC correlations from H-8 to C-11, from H-13 to C-9, and from CH_3_-10 to C-8, C-9, and C-11 revealed the existence of the five-membered ring C. Furthermore, the HMBC interactions from H-7 to C-14 and from CH_3_-15 and CH_3_-16 to C-7, C-13, and C-14 established the infrequent four-membered ring (ring D).

The relative configuration of **1** was illustrated by analyzing the NOESY interactions (Figure 2). The cross-peaks of H-7/H-8, H-7/CH3-15, CH_3_-15/H-13, and H-8/CH_3_-10 indicated that these protons were cofacial. However, the relative configuration of C-2’ could not be determined due to the absence of conclusive evidence. By meticulously comparing the 1D and 2D NMR data of **1** and **2**, almost identical chemical shifts and correlation signals were observed, except for the tiny difference presented at C-2′ and its surrounding signals. Herein, **1** and **2** were deduced to have the same planning structure as well as the same relative configuration in the 6/6/5/4 core, and were proposed to be a pair of C-2′ epimers (Figure 1).

**Figure 3:**
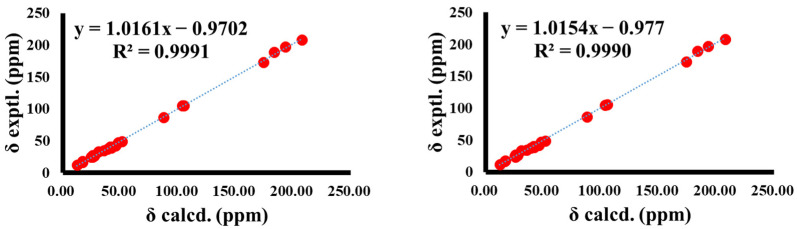
Regression analysis of the experimental (exptl.) versus calculated (calcd.) ^13^C NMR chemical shifts of **1** (**left**) and **2** (**right**).

To further confirm the novel 6/6/5/4 tetracyclic architecture in **1** and **2**, as well as try to determine the relative configuration of C-2′, the quantum chemical calculation of the ^13^C NMR data of two isomers, (7R*, 8R*, 9R*, 13S*, 2′S*)-**1** and (7R*, 8R*, 9R*, 13S*, 2′R*)-**2**, were performed. The results showed that both (7R*, 8R*, 9R*, 13S*, 2′S*)-**1** and (7R*, 8R*, 9R*, 13S*, 2′R*)-**2** have a good correlation coefficient (R_2_ ≥ 0.9990) with the experimental ^13^C NMR data of **1** and **2**, which solidified the deduced skeleton of **1** and **2** (Figure 3). Furthermore, the results confirmed that the change in configuration at C-2′ had a slight effect on the NMR data. Thus, the configuration of C-2’ in 2-methylbutanoyl fragment was difficult to assign by NMR calculations.

**Figure 4:**
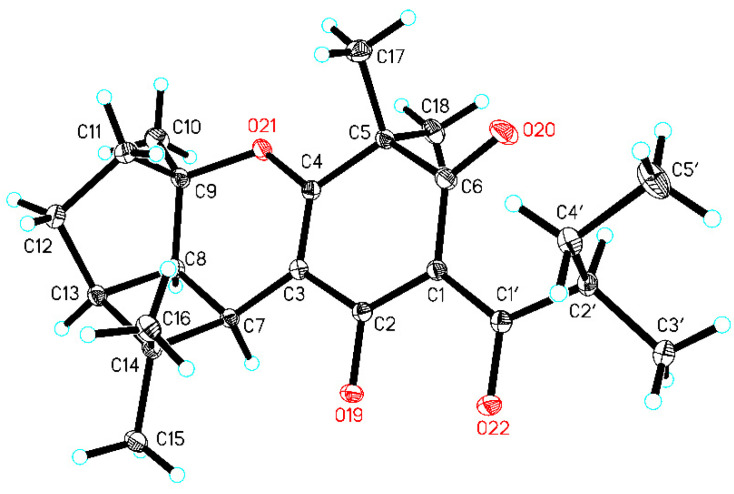
X-ray crystallographic structure of **2b**.

**Figure 5:**
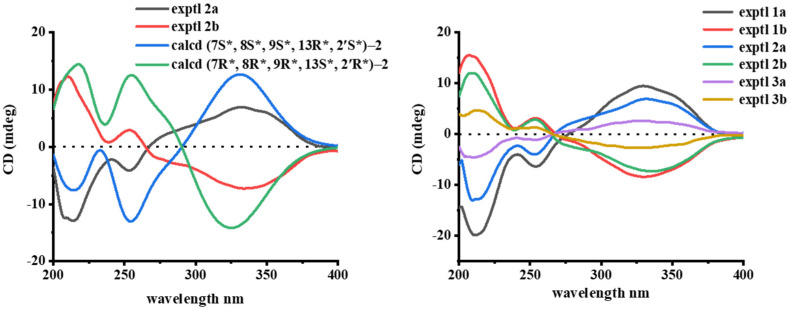
The experimental (exptl) and calculated (calcd) ECD spectra of **1a/b**–**3a/b**.

The application of single-crystal X-ray crystallography technique is an unambiguous method to determine the configuration of C-2’ in 2-methylbutanoyl fragment. Fortunately, a high-quality single crystal of **2b** was successfully obtained in a mixed solvent (acetone–water, 9:1) for X-ray diffraction. The results afforded fatal evidence for the absolute configuration of **2b** at C-2′, with a Flack parameter of 0.01(7) (CCDC 2080724) (Figure 4). Therefore, the absolute stereochemistry of **2b** was explicitly assigned as 7*R*, 8*R*, 9*R*, 13*S*, 2′*R*. The absolute configuration of **2a** could be accordingly assigned as 7*S*, 8*S*, 9*S*, 13*R*, 2′*S*, since **2a**/**2b** were a pair of enantiomers. Moreover, the quantum chemical ECD calculation method was used to further confirm the absolute configuration of **2a** and **2b**. The theoretically calculated ECD data of (7S*, 8S*, 9S*, 13R*, 2′S*)-**2** and (7R*, 8R*, 9R*, 13S*, 2′R*)-**2** were in good agreement with the experimental ECD data of **2a** and **2b**, which supported the assignment of the absolute configurations of **2a** and **2b** as 7*S*, 8*S*, 9*S*, 13*R*, 2′*S* and 7*R*, 8*R*, 9*R*, 13*S*, 2′*R*, respectively (Figure 5). Accordingly, the absolute configurations of **1a**/**1b** were respectively assigned as 7*S*, 8*S*, 9*S*, 13*R*, 2′*R* and 7*R*, 8*R*, 9*R*, 13*S*, 2′*S* by comparing their experimental ECD curves and NMR data with the C-2′ epimers **2a**/**2b** (Figure 5).

(±)-Hypersines C (**3a**/**3b**) were also obtained as a pair of enantiomers with the molecular formula of C_22_H_30_O_4_, based on the HR-ESI-MS data (m/z 359.2212, [M + H]^+^) (calculated for C_22_H_31_O_4_^+^, 359.2217). Extensive analysis of the ^1^H NMR and ^13^C NMR data of **3** and **2** suggested that **3** was the homologue of **2**, except for the absence of a methylene group. The ^1^H-^1^H COSY correlations of H-2’/H-3’/H-4’, together with the HMBC correlations from H-2’ to C-1’, from H-3’ to C-1’, and from H-4’ to C-1’, indicated the presence of an isobutyryl in **3** instead of a 2-methylbutanoyl group in **2**. The relative configuration of **3** was deduced by analyzing of the NOESY spectrum. The NOESY interactions of H-7/H-8, H-7/CH_3_-15, CH_3_-15/H-13, and H-8/CH_3_-10 suggested that the relative configuration of **3** was identical to that of **2**. The enantiomers **3a** and **3b** were successfully separated by chiral column (Figure S2). The overall pattern of the experimental ECD curve of **3a** and **3b**, correspondingly, have the same cotton effects with **2a** and **2b**, suggesting that they have the same absolute configurations. Meanwhile, the ECD data of **3b** were consistent with **2b**. Thus, the absolute configurations of **3a** and **3b** were assigned as 7*S*, 8*S*, 9*S*, 13*R* and 7*R*, 8*R*, 9*R*, 13*S*, respectively (Figure 5).

### 3.3. Plausible Biosynthetic Pathway to ***1**–**3***

**Scheme 1:**
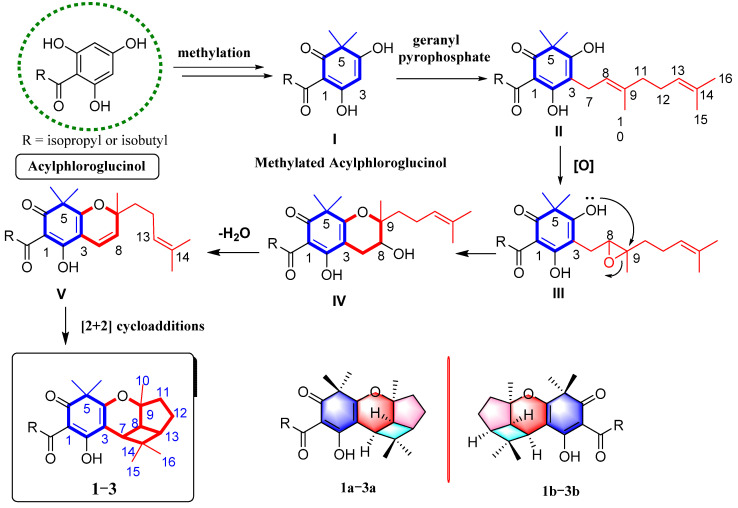
Plausible Biosynthetic Pathway to **1**–**3**.

(±)-Hypersines A–C represent the first example of MTPAPs with a rare 6/6/5/4 ring system, which is quite different from the known MTPAPs. The plausible biosynthetic pathway for **1**–**3** was proposed (Scheme 1). Acylphloroglucinol was considered as the biogenetic precursor to PPAPs, which had undergone methylation reaction to obtain intermediate **I** with a filicinic acid core. Subsequently, intermediate **II** could be formed by the enzyme-catalyzed addition of geranyl pyrophosphate to the filicinic core. Then, epoxidation occurred at Δ [8] to generate an epoxypropane intermediate **III**. Next, the six-membered oxygen ring was formed and generated intermediate **IV**. After the dehydration of OH-8, the unsaturated pyranoid ring was generated. Finally, the 6/6/5/4 tetracyclic skeleton was connected by [2 + 2] cycloadditions [9,19].

### 3.4. Biological Activity Evaluation of the Isolated Compounds

**Figure 6:**
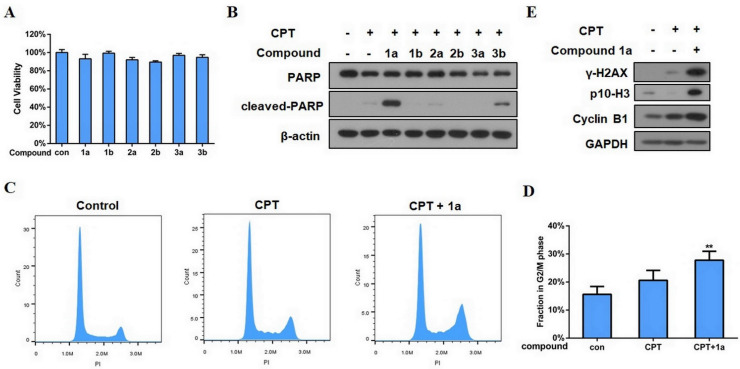
(**A**) MCF-7 cells were exposed to compounds (**1a**–**3b**) for 48 h. The cells viability was measured via MTT assay. The group for con means MCF-7 cells only treated with DMSO. (**B**) MCF-7 cells were incubated with 50 nM CPT, with or without 20 μM compounds, for 48 h, western blotting was used to analyze the level of cleaved-PARP and PARP. (**C**) After treatment with CPT plus **1a** for 48 h, the cell cycle of MCF-7 cells was determined through flow cytometry. (**D**) The percentages of G2/M phase. The group for con means MCF-7 cells only exposed to DMSO. ** *p* < 0.01vs Con group. (**E**) MCF-7 cells were treated with CPT and **1a** in combination for 48 h, the levels of relative protein (p10-H3, cyclin B1, γ-H2AX) were investigated by western blotting.

The biological activities of the isolated compounds, (±)-Hypersines A–C (**1a**–**3b**), were evaluated. In our previous studies, several PPAPs were found to show an inhibitory effects on the proliferation of human breast cancer cells (MCF-7) [6,21]. In this study, the MTT assay was used to measure the viability of MCF-7 cells after exposure to the isolated compounds. The results showed that none of the compounds could suppress the proliferation of MCF-7 cells in 20 μM (Figure 6A). Poly ADP-ribose polymerase (PARP) is a marker protein of apoptosis. Interestingly, when the compounds were co-treated with the anticancer agent, camptothecin (CPT), in a low concentration, **1a** (20 μM) could visibly trigger the cleavage of PARP combined with CPT (50 nM), while there was no PARP cleavage when treated with CPT (50 nM) alone (Figure 6B). As known, CPT causes cell death by suppressing DNA synthesis and eliciting DNA double-strand break (DSB) [31]. The above result indicated that **1a** could reinforce the CPT-induced apoptosis of MCF-7 cells. Further research exhibited that the percentage of MCF-7 cells in the G2/M phase increased after the co-treatment of CPT and **1a** (Figure 6C or Figure 6D). Meanwhile, the combination of CPT and **1a** obviously augmented the expression of γ-H2AX (the biomarker of DSB) (Figure 6E) [32]. Furthermore, the CPT restrained the phosphorylation of the Ser 10 of histone H3 (pS10-H3, the mitotic biomarker) [33]. This may be owing to CPT-induced DSB causing DNA damage response (DDR), which means that cells with damaged DNA cannot prematurely undergo mitosis [34]. However, synergy between CPT and **1a** could strongly elevate the level of pS10-H3, indicating that the majority of cells might enter M phase, which is demonstrated by the increasing level of Cyclin B1 (Figure 6E) [33]. Furthermore, the flow cytometry analysis indicated that the cells were arrested at G2/M phase with the co-treatment of CPT and **1a** (Figure 6C or Figure 6D). The above data indicated that **1a** might urge MCF-7 cells with CPT-induced DSB to progress into mitosis by restricting DDR and by rendering damaged DNA to remain in M phase. Compromised DNA entering into mitosis can lead to the apoptosis of cells [35]. This may be the reason why **1a** potentiated the CPT-induced apoptosis of MCF-7 cells, but specific mechanisms remained to be investigated. DDR inhibitors are usually used as sensitizers of antitumor agents to play a role in tumor treatment [36]. Therefore, **1a** might be considered as a novel inhibitor of DDR, and could enhance the sensitivity of MCF-7 cells to CPT.

## 4. Conclusions

In summary, (±)-hypersines A–C (**1**–**3**), the three pairs of unprecedented MPAPs with a rare 6/6/5/4 tetracyclic scaffold, were obtained from *H. elodeoides*. As far as we know, hypersines A–C are the first reported MTPAPs characterized by a peculiar 6/6/5/4 tetracyclic core, formed by a rare, tensional four-membered carbocycle concurrently fused with five- and six-membered rings. Furthermore, the possible biosynthetic pathway of **1**–**3** was proposed, which might provide a reference for the organic synthesis of these compounds. The biological activity results showed that the combination of **1a** and a low concentration of CPT could visibly trigger the cleavage of PARP, and could up-regulate the expression level of γ-H2AX, pS10-H3, and Cyclin B1, indicating that **1a** might enhance MCF-7 cell sensitivity to camptothecin by inhibiting the DNA damage response. This is the first report of MTPAPs as potential sensitizers of anti-tumor drugs. In general, this study enriches the structural diversity and biological activity of MTPAPs, and provides novel insight for further understanding MTPAPs. However, among these compounds, only **1a** exhibited potent activity. In future research, more analogues need to be obtained to further investigate the structure–activity relationship. A more detailed mechanism that **1a** enhances MCF-7 cell sensitivity to CPT needs to be further explored. This research suggests that the co-administration of **1a** and chemotherapeutic drugs could be an effective strategy for overcoming tumor MDR, and this deserves further exploration.

## Figures and Tables

**Table 1:** ^1^H NMR (600 MHz, in CDCl_3_) and ^13^C NMR (150 MHz, in CDCl_3_) data for **1**–**3**.

No.	1a/1b		2a/2b		3a/3b	
	*δ*_H_ (mult, *J*, Hz)	*δ*_C_	*δ*_H_ (mult, *J*, Hz)	*δ*_C_	*δ*_H_ (mult, *J*, Hz)	*δ*_C_
1		105.6		105.5		104.9
2		189.4		189.3		189.3
3		105.0		105.0		104.8
4		172.7		172.7		172.8
5		48.8		48.8		48.7
6		197.3		197.3		197.2
7	2.90 d (9.6)	34.8	2.90 d (9.6)	34.8	2.90 d (9.6)	34.8
8	2.55 dd (9.6, 7.2)	37.6	2.55 t (7.8)	37.6	2.55 t (7.2)	37.5
9		86.7		86.7		86.7
10	1.40 s	27.4	1.40 s	27.4	1.40 s	27.4
11	*α* 1.77 m	40.4	*α* 1.76 m	40.4	*α* 1.76 m	40.3
	*β* 1.88 m		*β* 1.88 m		*β* 1.88 m	
12	*α* 1.61 m	26.2	*α* 1.61 m	26.2	*α* 1.61 m	26.1
	*β* 1.70 m		*β* 1.70 m		*β* 1.68 m	
13	2.42 t (7.8)	47.1	2.42 t (7.2)	47.1	2.43 t (7.8)	47.0
14		38.8		38.8		38.8
15	1.34 s	33.5	1.35 s	33.5	1.34 s	33.5
16	0.80 s	18.1	0.80 s	18.0	0.80 s	18.0
17	1.32 s	24.5	1.32 s	24.1	1.32 s	24.3
18	1.36 s	25.2	1.36 s	25.6	1.36 s	25.5
1’		207.9		208.0		208.6
2’	3.84 m	42.1	3.83 m	42.2	3.97 m	35.9
3’	1.14 d (7.2)	16.7	1.12 d (7.2)	16.7	1.13 d (7.2)	19.1
4’	*α* 1.40 overlap	26.9	*α* 1.41 overlap	26.8	1.15 d (7.2)	19.2
	*β* 1.73 m		*β* 1.74 m			
5’	0.90 t (7.2)	11.9	0.93 t (7.2)	12.0		

## Supplementary Materials

The following are available online at https://www.mdpi.com/article/10.3390/biomedicines9101473/s1, Figure S1: MTPAPs derivatives reported previously.
