# Supplementary material for: Unprecedented Monoterpenoid Polyprenylated Acylphloroglucinols with a Rare 6/6/5/4 Tetracyclic Core, Enhanced MCF-7 Cells’ Sensitivity to Camptothecin by Inhibiting the DNA Damage Response
Source: Biomedicines. 2021 Oct 14;9(10):1473. doi: 10.3390/biomedicines9101473 (PMC8533472; doi:10.3390/biomedicines9101473)

Unprecedented monoterpenoid polyprenylated acylphloroglucinols with a rare 6/6/5/4 tetracyclic core, enhanced MCF-7 cells sensitivity to camptothecin by inhibiting the DNA damage response

Xiang-Zhong Liu, [†,‖] *Mi Zhou,* [†,‖] *Chun-Chun Du,*[†] *Hong-Hong Zhu,*[†] *Xi Lu,*[†] *Shou-Lun He,*[†] *Guang-Hui Wang,*[†] *Ting Lin,* [†] *Rong Ding,*[†] *Cui-ling Sun,*[†] *Wen-Jing Tian,*[*,†] *Hai-Feng Chen* [*,†]

[†]Fujian Provincial Key Laboratory of Innovative Drug Target, School of Pharmaceutical Sciences, Xiamen University, Xiamen 361102, People's Republic of China.

List of Supporting Information

1. Previously reported MTPAPs 1-5
2. The HPLC chromatogram and ^{1}H NMR data of **1** − **3** 6
3. Computational details 6-19
4. Spectral information of **1** 20-25
4.1 HR-ESI-MS, IR and UV (CH_3OH) spectrum of **1b** 20-21
4.2 1D and 2D NMR spectra of **1** in $CDCl_3$ 22-25
4.2.1 ^{1}H NMR spectrum of **1a** in $CDCl_3$ 22
4.2.2 ^{1}H NMR spectrum of **1b** in $CDCl_3$ 22
4.2.3 ^{13}C NMR spectrum of **1b** in $CDCl_3$ 23
4.2.4 DEPT-135 spectrum of **1b** in $CDCl_3$ 23
4.2.5 ^{1}H–^{1}H COSY spectrum of **1b** in $CDCl_3$ 24
4.2.6 HSQC spectrum of **1b** in $CDCl_3$ 24
4.2.7 HMBC spectrum of **1b** in $CDCl_3$ 25
4.2.8 NOESY spectrum of **1b** in $CDCl_3$ 25
5. Spectral information of **2** 26-31
5.1 HR-ESI-MS, IR and UV (CH_3OH) spectrum of **2a** 26-27
5.2 1D and 2D NMR spectra of **2** in $CDCl_3$ 28-31
5.2.1 ^{1}H NMR spectrum of **2a** in $CDCl_3$ 28
5.2.2 ^{1}H NMR spectrum of **2b** in $CDCl_3$ 28
5.2.3 ^{13}C NMR spectrum of **2a** in $CDCl_3$ 29
5.2.4 DEPT-135 spectrum of **2a** in $CDCl_3$ 29
5.2.5 ^{1}H–^{1}H COSY spectrum of **2a** in $CDCl_3$ 30
5.2.6 HSQC spectrum of **2a** in $CDCl_3$ 30
5.2.7 HMBC spectrum of **2a** in $CDCl_3$ 31
5.2.8 NOESY spectrum of **2a** in $CDCl_3$ 31
6. Spectral information of **3a** 32-37
6.1 HR-ESI-MS, IR and UV (CH_3OH) spectrum of **3a** 32-33
6.2 1D and 2D NMR spectra of **3a** in $CDCl_3$ 34-37
6.2.1 ^{1}H NMR spectrum of **3a** in $CDCl_3$ 34
6.2.2 ^{13}C NMR spectrum of **3a** in $CDCl_3$ 34
6.2.3 DEPT-135 spectrum of **3a** in $CDCl_3$ 35
6.2.4 ^{1}H–^{1}H COSY spectrum of **3a** in $CDCl_3$ 35
6.2.5 HSQC spectrum of **3a** in $CDCl_3$ 36
6.2.6 HMBC spectrum of **3a** in $CDCl_3$ 36
6.2.7 NOESY spectrum of **3a** in $CDCl_3$ 37

1. Previously reported MTPAPs

In the past decades, a series of polycyclic polyprenylated acylphloroglucinols (PPAPs) have been reported isolating from the plants of genera *Hypericum* and *Garcinia*. Monoterpenoid polyprenylated acylphloroglucinols (MTPAPs), a special type of structurally diverse PPAPs, generally decorated with a geranyl or a cyclic monoterpene fragment at C-3 of the phloroglucinol ring. To date, approximately 110 MTPAPs (Table S1) have been identified, which can be divided into six types due to their different ring system, including the uncyclized MTPAPs and cyclized MTPAPs with 6/5, 6/6, 6/5/6, 6/6/6 and 6/7/5 ring system.[1-24] Most of them were obtained from the genus *Hypericum*. Our work on *Hypericum elodeoides* have found three pairs of novel scaffolds MTPAPs, (±)-Hypersines A – C (**1**–**3**), which were characterized by an unusual large tensional four-membered carbocycle concurrently merged with five- and six-membered rings in the part of monoterpene fragment.

Table S1. Previously Reported MTPAPs

No.	Names	No.	Names
1-2	chinesin I−II[1]	34-41	tomoeones A−H[9]
3-4	hypercalin B−C[2]	42-45	(±)-japonicols A−D[10]
5-6	isomeric homologues of hypercalin C (5a/5b)[2]	46-47	hyperpatulone E−F[11]
7	paglucinol[3]	48-56	hyperpatulols A−I[12]
8-9	yojironins C−D[4]	57-68	hyperbeanols F−Q[13]
10-11	empetrikarinens A−B[5]	69-74	faberiones A−F[14]
12-13	empetrikarinols A−B[5]	75-76	hyphenrone J−K[15]
14-15	empetriferdinan A−B[5]	77-81	hypascyrin A−E[16]
16	empetriferdinol[5]	82	ent-hyphenrone J[16]
17-18	empetrifranzinan A/B[5]	83	hypascyrin K[16]
19	empetrifranzinan C[5]	84-85	hyperpatulone A−B[17]
20	madeleinol A[6]	86-87	spirohypatone A−B[18]
21	empetrifranzinan D[6]	88-89	hypatone A−B[19]
22	madeleinol B[6]	90-94	hyperhenols A−E[20]
23	3-geranyl-2,4,6-trihydroxybenzophenone[7]	95	longisglucinols B−C[21]
24	3-geranyl-1-(2'-methylpropanoyl)-phloroglucinol[8]	96-100	bellumone F−J[22]
25	3-geranyl-1-(2'-methylbutanoyl)-phloroglucinol[8]	101-103	hyperelodione A−C[23]

26-33	hyperascyrone A－H[9]	104-111	elodeoidols A－I[24]

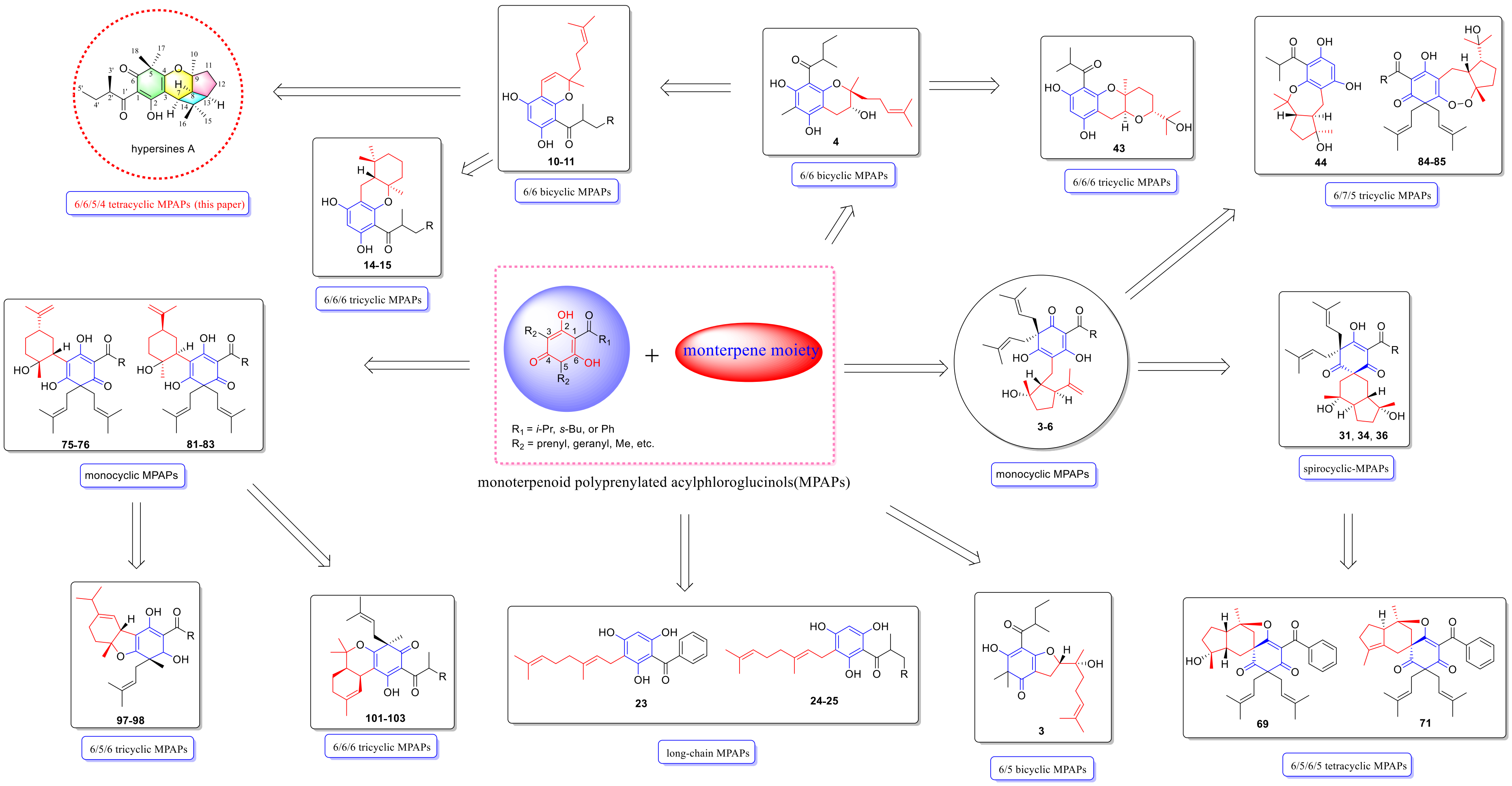

Figure S1. MTPAPs derivatives reported previously

Reference

1. M, N.; M, T., Antimicrobial compounds, chinesin I and II from flowers of *Hypericum chinese* L. *Chemistry Letters* **1987**, 1337-1340.

2. Decosterd, L. A.; Stoeckli-Evans, H.; Chapuis, J.-C.; Sorda, B.; Hostettmann, K., New Cell Growth-Inhibitory Cyclohexadienone Derivatives from *Hypericum calycinum* L. *Helvetica Chimica Acta* **1989,** *72*, 1833-1845.

3. Ishiguro, K.; Nagareya, N.; Fukumoto, H., A phloroglucinol derivative from cell suspension cultures of *Hypericum patulum*. *Phytochemistry* **1998,** *47*, 1041-1043.

4. Mamemura, T.; Tanaka, N.; Shibazaki, A.; Gonoi, T.; Kobayashi, J. i., Yojironins A−D, meroterpenoids and prenylated acylphloroglucinols from *Hypericum yojiroanum*. *Tetrahedron Letters* **2011,** *52*, 3575-3578.

5. Schmidt, S.; Jurgenliemk, G.; Schmidt, T. J.; Skaltsa, H.; Heilmann, J., Bi-, tri-, and polycyclic acylphloroglucinols from *Hypericum empetrifolium*. *J Nat Prod* **2012,** *75*, 1697-705.

6. Fobofou, S. A.; Franke, K.; Sanna, G.; Porzel, A.; Bullita, E.; La Colla, P.; Wessjohann, L. A., Isolation and anticancer, anthelminthic, and antiviral (HIV) activity of acylphloroglucinols, and regioselective synthesis of empetrifranzinans from *Hypericum roeperianum*. *Bioorg Med Chem* **2015,** *23*, 6327-34.

7. Zhang, Z.; Elsohly, H. N.; Jacob, M. R.; Pasco, D. S.; Walker, L. A.; Clark, A. M., Natural Products Inhibiting Candida albicans Secreted Aspartic Proteases from *Tovomita krukovii*. *Planta Medica* **2002,** *68*, 49-54.

8. Crockett, S. L.; Wenzig, E. M.; Kunert, O.; Bauer, R., Anti-inflammatory phloroglucinol derivatives from *Hypericum empetrifolium*. *Phytochem Lett* **2008,** *1*, 37-43.

9. Zhu, H.; Chen, C.; Liu, J.; Sun, B.; Wei, G.; Li, Y.; Zhang, J.; Yao, G.; Luo, Z.; Xue, Y.; Zhang, Y., Hyperascyrones A-H, polyprenylated spirocyclic acylphloroglucinol derivatives from *Hypericum ascyron* Linn. *Phytochemistry* **2015,** *115*, 222-30.

10. Hu, L.; Xue, Y.; Zhang, J.; Zhu, H.; Chen, C.; Li, X. N.; Liu, J.; Wang, Z.; Zhang, Y.; Zhang, Y., (+/-)-Japonicols A-D, Acylphloroglucinol-Based Meroterpenoid Enantiomers with Anti-KSHV Activities from *Hypericum japonicum*. *J Nat Prod* **2016,** *79*, 1322-8.

11. Wu, Z.-N.; Niu, Q.-W.; Zhang, Y.-B.; Luo, D.; Li, Q.-G.; Li, Y.-Y.; Kuang, G.-K.; He, L.-J.; Wang, G.-C.; Li, Y.-L., Hyperpatulones A–F, polycyclic polyprenylated acylphloroglucinols from *Hypericum patulum* and their cytotoxic activities. *RSC Advances* **2019,** *9*, 7961-7966.

12. Liu, Y. Y.; Ao, Z.; Xu, Q. Q.; Zhu, D. R.; Chen, C.; Wang, X. B.; Luo, J. G.; Kong, L. Y., Hyperpatulols A-I, spirocyclic acylphloroglucinol derivatives with anti-migration activities from the flowers of *Hypericum patulum*. *Bioorg Chem* **2019,** *87*, 409-416.

13. Li, Y. R.; Xu, W. J.; Wei, S. S.; Lu, W. J.; Luo, J.; Kong, L. Y., Hyperbeanols F-Q, diverse monoterpenoid polyprenylated acylphloroglucinols from the flowers of *Hypericum beanii*. *Phytochemistry* **2019,** *159*, 56-64.

14. Zhang, X. W.; Fan, S. Q.; Xia, F.; Ye, Y. S.; Yang, X. W.; Yang, X. W.; Xu, G., Prenylated Acylphloroglucinols from *Hypericum faberi*. *J Nat Prod* **2019,** *82*, 1367-1371.

15. Yang, X. W.; Li, M. M.; Liu, X.; Ferreira, D.; Ding, Y.; Zhang, J. J.; Liao, Y.; Qin, H. B.; Xu, G., Polycyclic Polyprenylated Acylphloroglucinol Congeners Possessing Diverse Structures from *Hypericum henryi*. *J Nat Prod* **2015,** *78*, 885-95.

16. Niwa, K.; Tanaka, N.; Tatano, Y.; Yagi, H.; Kashiwada, Y., Hypascyrins A-E, Prenylated

Acylphloroglucinols from *Hypericum ascyron*. *J Nat Prod* **2019,** *82*, 2754-2760.
17. Ao, Z.; Liu, Y.-Y.; Lin, Y.-L.; Chen, X.-l.; Chen, K.; Kong, L.-Y.; Luo, J.-G., Hyperpatulones A and B, two new peroxide polyprenylated acylphloroglucinols from the leaves of *Hypericum patulum*. *Tetrahedron Letters* **2020,** *61*.
18. Ye, Y.; Jiang, N.; Yang, X.; Xu, G., Polycyclic polyprenylated acylphloroglucinol with an unprecedented spirocyclic core from *Hypericum patulum*. *Chinese Chemical Letters* **2020,** *31*, 2433-2436.
19. Ye, Y. S.; Li, W. Y.; Du, S. Z.; Yang, J.; Nian, Y.; Xu, G., Congenetic Hybrids Derived from Dearomatized Isoprenylated Acylphloroglucinol with Opposite Effects on Cav3.1 Low Voltage-Gated Ca(2+) Channel. *J Med Chem* **2020,** *63*, 1709-1716.
20. Ye, Y. S.; Wu, M.; Jiang, N. N.; Lao, Y. Z.; Fu, W. W.; Liu, X.; Yang, X. W.; Zhang, J.; Xu, H. X.; Xu, G., Dearomatized Isoprenylated Acylphloroglucinol Derivatives with Potential Antitumor Activities from *Hypericum henryi*. *Nat Prod Bioprospect* **2020,** *10*, 1-11.
21. Zhang, N.; Shi, Z.; Xu, Q.; Sun, W.; Gu, L.; Xie, S.; Guo, Y.; Duan, Y.; Zhang, K.; Qi, C.; Zhang, Y., Longisglucinols A-C, Structurally Intriguing Polycyclic Polyprenylated Acylphloroglucinols with Anti-inflammatory Activity from *Hypericum longistylum*. *Org Lett* **2020,** *22*, 7926-7929.
22. Zhou, X.; Xu, W.; Li, Y.; Zhang, M.; Tang, P.; Lu, W.; Li, Q.; Zhang, H.; Luo, J.; Kong, L., Anti-Inflammatory, Antioxidant, and Anti-Nonalcoholic Steatohepatitis Acylphloroglucinol Meroterpenoids from *Hypericum bellum* Flowers. *J Agric Food Chem* **2021,** *69*, 646-654.
23. Qiu, D.; Zhou, M.; Chen, J.; Wang, G.; Lin, T.; Huang, Y.; Yu, F.; Ding, R.; Sun, C.; Tian, W.; Chen, H., Hyperelodiones A-C, monoterpenoid polyprenylated acylphoroglucinols from *Hypericum elodeoides*, induce cancer cells apoptosis by targeting RXRalpha. *Phytochemistry* **2020,** *170*, 112216.
24. Li, Q. J.; Tang, P. F.; Zhou, X.; Lu, W. J.; Xu, W. J.; Luo, J.; Kong, L. Y., Dimethylated acylphloroglucinol meroterpenoids with anti-oral-bacterial and anti-inflammatory activities from *Hypericum elodeoides*. *Bioorg Chem* **2020,** *104*, 104275.

2. The HPLC chromatogram and ^{1}H NMR data of 1 – 3

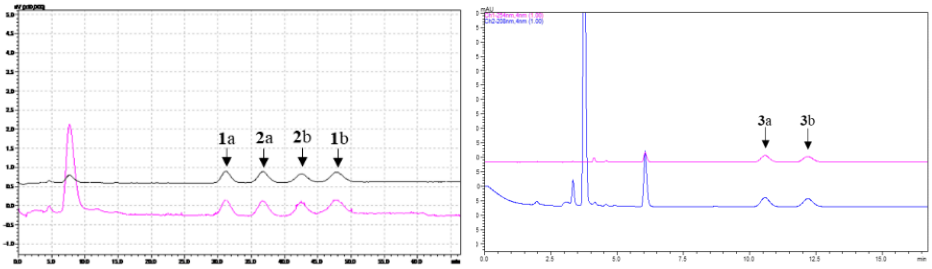

Figure S2. Chiral HPLC chromatogram of 1a/b−3a/b.

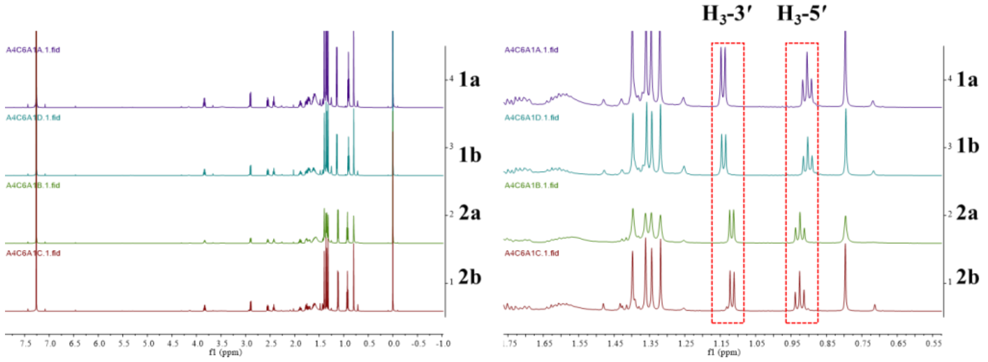

Figure S3. Comparing the ^{1}H NMR data of 1a/b and 2a/b (left) and partial enlarged detail(right).

3. Computational details

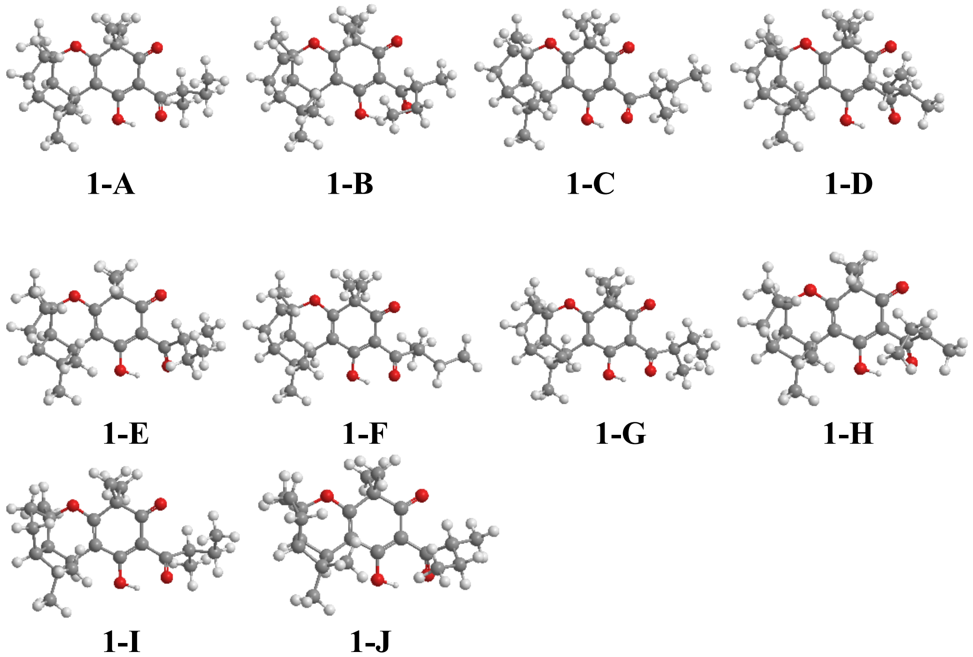

Figure S4. Conformers of isomer 1 for NMR calculation.

Table S3. Important thermodynamic parameters and Boltzmann distributions of the optimized isomer **1** at B3LYP/6-31G* level in the gas phase.

Conformations	Energy (a.u)	ΔG(kcal/mol)	%	Number of imaginary frequencies
1-A	-1196.706236	0	20.59%	0
1-B	-1196.706235	0.000188253	20.58%	0
1-C	-1196.705827	0.256337631	13.35%	0
1-D	-1196.705827	0.256400382	13.35%	0
1-E	-1196.705129	0.694527515	6.37%	0
1-F	-1196.705126	0.696410043	6.35%	0
1-G	-1196.704621	1.01323959	3.72%	0
1-H	-1196.704616	1.016377137	3.70%	0
1-I	-1196.704316	1.204378983	2.69%	0
1-J	-1196.704313	1.206575267	2.68%	0

Table S4. Optimized Z-matrixes of isomer **1** in the gas phase (Å) at B3LYP/6-31G* level.

**1**-A				**1**-B			
C	-10.3581	1.4893	2.6921	C	-10.0935	1.5916	2.5954
C	-10.2352	0.0081	2.2748	C	-9.8813	0.1163	2.1933
C	-10.1958	-0.2848	0.7671	C	-10.0896	-0.2342	0.7122
C	-10.7189	0.8757	-0.0877	C	-10.8942	0.824	-0.0512
C	-11.1341	2.0266	0.4915	C	-11.3236	1.9466	0.5713
O	-11.0992	2.3378	1.8194	O	-11.073	2.3197	1.8596
C	-10.8249	0.7186	-1.539	C	-11.2275	0.6062	-1.4593
C	-11.3234	1.6816	-2.3662	C	-11.9405	1.4936	-2.2106
C	-11.7199	2.9978	-1.8377	C	-12.4966	2.7169	-1.6069
C	-11.7028	3.2149	-0.2954	C	-12.1653	3.0287	-0.1174
C	-13.1766	3.4346	0.1446	C	-13.5208	3.1423	0.6374
C	-10.801	4.4544	-0.0218	C	-11.3585	4.3581	-0.1082
O	-12.0498	3.9208	-2.587	O	-13.2178	3.4829	-2.2513
C	-11.4599	1.3841	-3.8054	C	-12.1411	1.2144	-3.6465
O	-10.5648	0.7958	-4.4172	O	-12.3938	0.0748	-4.0463
C	-12.7906	1.681	-4.5412	C	-11.8795	2.3206	-4.6966
C	-13.7011	0.4332	-4.528	C	-13.1401	2.587	-5.5495
C	-12.5957	2.2097	-5.9876	C	-10.6524	2.0146	-5.5949
C	-11.8394	3.548	-6.0753	C	-9.3172	1.8921	-4.8354
O	-10.4058	-0.4733	-2.046	O	-10.7852	-0.5589	-2.0068
C	-8.9237	2.033	2.7864	C	-8.7375	2.2907	2.4082
C	-8.0331	0.8112	3.0371	C	-7.6826	1.1837	2.5101
C	-8.7548	-0.3937	2.4023	C	-8.367	-0.117	2.0463
C	-11.1184	1.6403	4.0195	C	-10.6094	1.7144	4.0381
C	-8.6909	-0.6448	0.8714	C	-8.5602	-0.4217	0.536
C	-8.4074	-2.1223	0.5285	C	-8.1767	-1.8718	0.1741
C	-7.7268	0.2512	0.0572	C	-7.8746	0.5412	-0.4633

H	-8.5666	-1.2817	3.0098	H	-7.9695	-0.9542	2.6245
H	-10.9434	-0.6108	2.8269	H	-10.3991	-0.5521	2.8823
H	-10.8094	-1.1521	0.5113	H	-10.6363	-1.1735	0.5965
H	-13.7949	2.5542	-0.0407	H	-14.0736	2.2009	0.6279
H	-13.2436	3.6545	1.211	H	-13.3742	3.4175	1.6827
H	-13.6385	4.2758	-0.3764	H	-14.1698	3.9062	0.2045
H	-10.7864	4.714	1.0375	H	-10.401	4.2587	-0.6233
H	-11.1507	5.3434	-0.5507	H	-11.9054	5.1735	-0.5863
H	-9.7665	4.2807	-0.3242	H	-11.1425	4.686	0.9095
H	-13.3136	2.4607	-3.9907	H	-11.6573	3.2409	-4.1576
H	-14.6662	0.6455	-4.9893	H	-12.9788	3.4084	-6.2481
H	-13.902	0.0919	-3.511	H	-13.9919	2.8628	-4.9248
H	-13.2537	-0.399	-5.0747	H	-13.4289	1.7103	-6.132
H	-12.0817	1.4619	-6.5956	H	-10.8296	1.1033	-6.1707
H	-13.5738	2.3447	-6.4521	H	-10.5491	2.8111	-6.334
H	-11.7854	3.9016	-7.1052	H	-8.4876	1.7478	-5.5283
H	-12.3335	4.3208	-5.4846	H	-9.1084	2.7911	-4.2543
H	-10.8164	3.4574	-5.7081	H	-9.3158	1.0445	-4.1491
H	-10.4242	-0.3842	-3.009	H	-11.1766	-0.6123	-2.8906
H	-8.6481	2.5109	1.8478	H	-8.6964	2.7583	1.426
H	-8.8067	2.7927	3.5594	H	-8.5673	3.0885	3.1314
H	-7.9508	0.6477	4.1133	H	-7.3847	1.0735	3.5545
H	-7.0164	0.9435	2.6649	H	-6.774	1.4102	1.9509
H	-10.6429	1.0682	4.8159	H	-9.9341	1.2302	4.7432
H	-11.1581	2.6828	4.3356	H	-10.7091	2.7585	4.3349
H	-12.146	1.2901	3.9221	H	-11.5908	1.2514	4.1415
H	-8.5657	-2.3152	-0.5335	H	-8.5071	-2.1243	-0.8345
H	-7.3783	-2.394	0.7651	H	-7.0972	-2.0192	0.2165
H	-9.0594	-2.8002	1.0806	H	-8.632	-2.594	0.8527
H	-6.6892	0.0804	0.3437	H	-6.7893	0.4987	-0.3732
H	-7.8088	0.0421	-1.0099	H	-8.1269	0.2795	-1.4913
H	-7.9326	1.3118	0.1782	H	-8.1774	1.5763	-0.3268

Continued table S4.

1-C				1-D			
C	-10.354	1.496	2.7167	C	-10.0282	1.6417	2.579
C	-10.2348	0.0102	2.3156	C	-9.914	0.1372	2.2522
C	-10.1773	-0.2976	0.8114	C	-10.1636	-0.2756	0.7937
C	-10.682	0.8577	-0.0618	C	-10.9158	0.7884	-0.0142
C	-11.0985	2.0159	0.5011	C	-11.2662	1.9659	0.5538
O	-11.079	2.34	1.8263	O	-10.9677	2.3926	1.8149
C	-10.7676	0.6877	-1.5132	C	-11.2867	0.5181	-1.404
C	-11.2471	1.6454	-2.3591	C	-11.977	1.3978	-2.1861

C	-11.6549	2.9652	-1.8439	C	-12.4687	2.6708	-1.6297
C	-11.6511	3.1987	-0.3041	C	-12.0597	3.0561	-0.1772
C	-13.1276	3.4331	0.1199	C	-13.3743	3.3123	0.6156
C	-10.7439	4.4353	-0.0354	C	-11.1661	4.3234	-0.287
O	-11.9866	3.8789	-2.6032	O	-13.1969	3.4222	-2.2819
C	-11.3578	1.339	-3.8021	C	-12.2101	1.0595	-3.6082
O	-10.4959	0.6703	-4.379	O	-12.528	-0.0884	-3.9307
C	-12.6268	1.7345	-4.5941	C	-11.925	2.1073	-4.7211
C	-13.3435	0.484	-5.159	C	-12.3668	1.6299	-6.1293
C	-12.2924	2.772	-5.6976	C	-10.438	2.5574	-4.7041
C	-13.5316	3.4228	-6.3342	C	-10.1543	3.8032	-5.56
O	-10.3491	-0.5127	-1.9992	O	-10.9017	-0.6888	-1.9017
C	-8.9176	2.0323	2.8239	C	-8.6333	2.2433	2.3453
C	-8.0379	0.8078	3.0988	C	-7.6488	1.0782	2.4934
C	-8.7587	-0.3992	2.4667	C	-8.4192	-0.198	2.1023
C	-11.13	1.6646	4.0328	C	-10.5193	1.8707	4.0175
C	-8.6763	-0.6663	0.9394	C	-8.6507	-0.5641	0.6112
C	-8.3982	-2.149	0.6154	C	-8.3595	-2.0509	0.3183
C	-7.6955	0.2151	0.1292	C	-7.9217	0.3051	-0.4416
H	-8.5839	-1.2821	3.0857	H	-8.0675	-1.029	2.7178
H	-10.954	-0.599	2.8642	H	-10.4629	-0.4608	2.9806
H	-10.7931	-1.1635	0.5561	H	-10.7666	-1.1851	0.733
H	-13.5795	4.2712	-0.4148	H	-13.9879	4.0894	0.1554
H	-13.75	2.5546	-0.0611	H	-13.9886	2.4128	0.689
H	-13.2032	3.6657	1.183	H	-13.1695	3.6448	1.6341
H	-11.0825	5.3205	-0.5778	H	-11.6764	5.1417	-0.7993
H	-10.7384	4.7065	1.021	H	-10.8881	4.7003	0.6983
H	-9.7075	4.2514	-0.3251	H	-10.2388	4.1247	-0.8279
H	-13.3095	2.205	-3.8874	H	-12.5396	2.9742	-4.4861
H	-12.7437	-0.0137	-5.9231	H	-11.765	0.7893	-6.4781
H	-14.3011	0.7421	-5.6107	H	-12.2862	2.4254	-6.8695
H	-13.5521	-0.2473	-4.3764	H	-13.4097	1.3082	-6.1302
H	-11.6774	3.5719	-5.2776	H	-10.124	2.7888	-3.6841
H	-11.6823	2.3087	-6.476	H	-9.7962	1.7373	-5.033
H	-13.2405	4.1908	-7.0514	H	-9.1171	4.1219	-5.4515
H	-14.1434	2.6961	-6.8684	H	-10.324	3.6157	-6.6202
H	-14.1572	3.9002	-5.5789	H	-10.7879	4.6397	-5.262
H	-10.3603	-0.4371	-2.9643	H	-11.3151	-0.7709	-2.7732
H	-8.6272	2.4987	1.8841	H	-8.5746	2.6587	1.3408
H	-8.806	2.7992	3.5905	H	-8.4046	3.0633	3.0264
H	-7.9709	0.6548	4.1775	H	-7.3456	1.0017	3.5393
H	-7.0156	0.9302	2.7387	H	-6.7347	1.2185	1.9151
H	-10.6679	1.0978	4.8408	H	-9.8682	1.3811	4.7413

H	-11.1678	2.7104	4.338	H	-10.5495	2.9329	4.2607
H	-12.1583	1.3192	3.9259	H	-11.5268	1.4771	4.1523
H	-8.5439	-2.3519	-0.4466	H	-8.7178	-2.3325	-0.6728
H	-7.374	-2.4249	0.8684	H	-7.2903	-2.261	0.3569
H	-9.0619	-2.8169	1.1657	H	-8.848	-2.7099	1.037
H	-6.6631	0.0406	0.4318	H	-6.8397	0.2029	-0.359
H	-7.7642	-0.0049	-0.9366	H	-8.2008	0.007	-1.4526
H	-7.8961	1.2782	0.2363	H	-8.1619	1.3618	-0.3555

Continued table S4.

1-E				1-F				1-G			
C	-10.1369	1.5903	2.6744	C	-10.4744	1.411	2.7294	C	-10.3408	1.4807	2.7293
C	-9.9568	0.1025	2.3043	C	-10.2539	-0.0422	2.2583	C	-10.194	0.0049	2.3014
C	-10.1233	-0.2672	0.8226	C	-10.1183	-0.2652	0.7442	C	-10.1685	-0.2781	0.7916
C	-10.8698	0.8005	0.0138	C	-10.6504	0.9067	-0.0895	C	-10.7266	0.8763	-0.0498
C	-11.2868	1.9451	0.6033	C	-11.1547	2.0087	0.5131	C	-11.1571	2.0143	0.5422
O	-11.0663	2.3351	1.8921	O	-11.2117	2.2592	1.8532	O	-11.1094	2.3194	1.8713
C	-11.167	0.5658	-1.4001	C	-10.6691	0.8099	-1.5499	C	-10.8458	0.7266	-1.5012
C	-11.8421	1.4522	-2.1888	C	-11.1535	1.791	-2.3651	C	-11.3735	1.6826	-2.3217
C	-12.3856	2.6989	-1.6184	C	-11.6298	3.0666	-1.8025	C	-11.8111	2.9794	-1.7711
C	-12.0789	3.035	-0.1289	C	-11.7361	3.2031	-0.2544	C	-11.7619	3.1932	-0.2291
C	-13.4474	3.2063	0.5913	C	-13.248	3.3141	0.0841	C	-13.2285	3.3888	0.2466
C	-11.2326	4.3395	-0.1299	C	-10.9328	4.4759	0.1467	C	-10.8741	4.4466	0.0259
O	-13.0784	3.4674	-2.2895	O	-11.931	4.0184	-2.5264	O	-12.2051	3.8913	-2.5014
C	-12.0195	1.1455	-3.6263	C	-11.2116	1.5458	-3.8233	C	-11.5082	1.3938	-3.7699
O	-12.23	-0.0081	-4.0111	O	-10.2758	0.9833	-4.3985	O	-10.6608	0.7202	-4.3628
C	-11.7948	2.2394	-4.6976	C	-12.504	1.8703	-4.623	C	-12.7972	1.7879	-4.5355
C	-13.1078	2.5414	-5.459	C	-13.6986	1.0354	-4.0985	C	-13.4974	0.5556	-5.1564
C	-10.6339	1.8604	-5.655	C	-12.3237	1.7241	-6.1607	C	-12.58	2.9223	-5.5774
C	-10.167	3.018	-6.5534	C	-13.4845	2.3026	-6.9878	C	-11.6528	2.5946	-6.7645
O	-10.7316	-0.6177	-1.9118	O	-10.1855	-0.3473	-2.0787	O	-10.4068	-0.4552	-2.0136
C	-8.7541	2.2444	2.5253	C	-9.0765	2.0197	2.9242	C	-8.9164	2.0524	2.8091
C	-7.7385	1.1076	2.6835	C	-8.1393	0.833	3.1738	C	-7.9986	0.8471	3.0407
C	-8.4462	-0.1792	2.2161	C	-8.7639	-0.3771	2.4518	C	-8.7044	-0.368	2.4075
C	-10.7001	1.7539	4.0953	C	-11.3098	1.4659	4.0185	C	-11.0871	1.6088	4.067
C	-8.595	-0.5034	0.7048	C	-8.6055	-0.5587	0.9179	C	-8.6554	-0.6083	0.8743
C	-8.2429	-1.9702	0.3802	C	-8.2319	-2.0052	0.5316	C	-8.3466	-2.0777	0.5183
C	-7.8452	0.4215	-0.2838	C	-7.6441	0.4158	0.1959	C	-7.72	0.312	0.0537
H	-8.0959	-1.0182	2.8214	H	-8.5637	-1.2803	3.0325	H	-8.4907	-1.2559	3.0068
H	-10.5193	-0.5375	2.9851	H	-10.9585	-0.7188	2.7433	H	-10.8825	-0.6313	2.8588
H	-10.6933	-1.192	0.703	H	-10.6738	-1.148	0.4184	H	-10.7677	-1.1563	0.5388
H	-14.0631	3.9793	0.1268	H	-13.7213	4.1546	-0.4278	H	-13.2741	3.6061	1.3147
H	-14.0283	2.2819	0.5898	H	-13.7968	2.4112	-0.1907	H	-13.7165	4.2233	-0.2611

H	-13.3168	3.5012	1.6334	H	-13.4043	3.4716	1.1523	H	-13.8369	2.4989	0.0744
H	-11.0305	4.6837	0.8853	H	-11.0101	4.6798	1.2154	H	-11.2502	5.3305	-0.4932
H	-10.2668	4.2002	-0.6195	H	-9.8706	4.3776	-0.0856	H	-10.8396	4.7044	1.0852
H	-11.7433	5.1598	-0.6387	H	-11.2967	5.3707	-0.3624	H	-9.844	4.2899	-0.3001
H	-11.4941	3.1445	-4.1708	H	-12.7224	2.9207	-4.441	H	-13.4893	2.1774	-3.7896
H	-13.4332	1.6883	-6.0567	H	-13.5573	-0.0304	-4.2848	H	-14.4207	0.8403	-5.6619
H	-12.9951	3.3919	-6.1306	H	-14.6331	1.3307	-4.5748	H	-13.7609	-0.179	-4.394
H	-13.9209	2.7907	-4.775	H	-13.8478	1.1664	-3.0256	H	-12.8651	0.0518	-5.8892
H	-9.7731	1.5193	-5.0753	H	-11.4099	2.2377	-6.469	H	-13.5521	3.2277	-5.9674
H	-10.9255	1.0123	-6.279	H	-12.175	0.6748	-6.424	H	-12.195	3.8108	-5.0733
H	-9.3079	2.7207	-7.1557	H	-13.2635	2.251	-8.0544	H	-12.015	1.7402	-7.3361
H	-10.9495	3.3356	-7.2428	H	-14.4129	1.7555	-6.8248	H	-11.5877	3.4404	-7.4494
H	-9.8703	3.885	-5.962	H	-13.6637	3.3497	-6.7402	H	-10.6382	2.3691	-6.435
H	-11.0977	-0.6806	-2.8062	H	-10.1577	-0.2199	-3.0375	H	-10.4482	-0.3671	-2.9775
H	-8.6634	2.6935	1.5378	H	-8.7765	2.5504	2.0222	H	-8.6624	2.5416	1.8702
H	-8.5851	3.049	3.2413	H	-9.0395	2.7502	3.7326	H	-8.8047	2.8095	3.5855
H	-7.482	1.0064	3.7397	H	-8.106	0.6272	4.2453	H	-7.8994	0.6787	4.1147
H	-6.8038	1.2965	2.1542	H	-7.1124	1.0308	2.864	H	-6.9896	1.0019	2.6566
H	-10.066	1.2615	4.8322	H	-10.8476	0.8833	4.8153	H	-10.5904	1.0415	4.8539
H	-10.7776	2.8057	4.3712	H	-11.4197	2.4902	4.3749	H	-11.1435	2.6484	4.3901
H	-11.6986	1.3232	4.1704	H	-12.3115	1.07	3.851	H	-12.1087	1.2388	3.9803
H	-8.5447	-2.2301	-0.6355	H	-8.3234	-2.1592	-0.5445	H	-8.5148	-2.2672	-0.5428
H	-7.1705	-2.1488	0.4638	H	-7.2048	-2.2383	0.814	H	-7.3092	-2.3299	0.74
H	-8.7435	-2.667	1.0535	H	-8.8786	-2.736	1.0185	H	-8.9776	-2.772	1.0743
H	-6.7655	0.3486	-0.1534	H	-6.6162	0.2804	0.5319	H	-6.6757	0.161	0.3266
H	-8.0679	0.1492	-1.3159	H	-7.6593	0.2501	-0.8819	H	-7.8107	0.1071	-1.0135
H	-8.1216	1.4672	-0.1755	H	-7.9071	1.4594	0.3494	H	-7.9462	1.3674	0.1833

Continued table S4.

1-H				1-I				1-J			
C	-10.0278	1.6192	2.5466	C	-10.5583	1.3543	2.7642	C	-10.2502	1.4059	2.7188
C	-9.8493	0.1347	2.1626	C	-10.1169	-0.0385	2.2644	C	-9.7393	0.0672	2.1435
C	-10.1123	-0.2371	0.6954	C	-10.1146	-0.3135	0.7517	C	-9.9927	-0.243	0.6594
C	-10.9274	0.82	-0.0585	C	-10.677	0.8367	-0.0866	C	-10.8494	0.8052	-0.0536
C	-11.3201	1.9584	0.5591	C	-10.9864	2.0212	0.485	C	-11.1907	1.956	0.5662
O	-11.0219	2.3485	1.832	O	-10.8991	2.3516	1.8025	O	-10.8893	2.3303	1.8395
C	-11.3091	0.5851	-1.4514	C	-10.8601	0.6603	-1.5287	C	-11.2574	0.5825	-1.4417
C	-12.0334	1.4706	-2.1947	C	-11.3031	1.6478	-2.3583	C	-11.9297	1.5058	-2.1865
C	-12.5514	2.7114	-1.5926	C	-11.5615	3.0025	-1.8436	C	-12.3744	2.7763	-1.5897
C	-12.1673	3.0418	-0.1199	C	-11.4872	3.2378	-0.3058	C	-11.9867	3.0792	-0.1122
C	-13.4956	3.1887	0.6762	C	-12.9287	3.5729	0.1673	C	-13.3139	3.2785	0.6744
C	-11.34	4.3581	-0.1592	C	-10.4883	4.4099	-0.0738	C	-11.1033	4.3592	-0.1362
O	-13.2785	3.4809	-2.2252	O	-11.8239	3.9408	-2.6005	O	-13.0497	3.5873	-2.2289

C	-12.2767	1.1722	-3.6233	C	-11.5246	1.3367	-3.7841	C	-12.2024	1.2152	-3.6077
O	-12.5724	0.0281	-3.9791	O	-10.7145	0.6538	-4.4158	O	-12.5625	0.0929	-3.9723
C	-12.0279	2.2617	-4.7048	C	-12.8495	1.7449	-4.476	C	-11.8853	2.2748	-4.69
C	-12.3041	1.7751	-6.1502	C	-13.8727	0.5902	-4.4001	C	-13.1418	2.6123	-5.5236
C	-10.6305	2.9364	-4.5946	C	-12.6629	2.2247	-5.9405	C	-10.7029	1.86	-5.6044
C	-9.4181	1.9898	-4.7016	C	-11.7871	3.4824	-6.0874	C	-9.364	1.6586	-4.8688
O	-10.8988	-0.594	-1.9936	O	-10.5701	-0.5741	-2.0244	O	-10.9249	-0.6234	-1.979
C	-8.6697	2.2961	2.3016	C	-9.3118	1.8884	3.4798	C	-8.9677	2.1066	3.1827
C	-7.6269	1.1764	2.387	C	-8.1385	1.3494	2.6592	C	-7.9138	1.6992	2.1511
C	-8.3441	-0.1221	1.9688	C	-8.5783	-0.0796	2.2816	C	-8.2334	0.2184	1.8624
C	-10.4924	1.7732	4.0037	C	-11.8015	1.3124	3.6637	C	-11.2852	1.2323	3.8391
C	-8.5923	-0.4491	0.4713	C	-8.5862	-0.5838	0.8169	C	-8.4585	-0.316	0.4259
C	-8.2408	-1.9104	0.1219	C	-8.2445	-2.0853	0.7113	C	-7.954	-1.7643	0.2523
C	-7.9279	0.4872	-0.5668	C	-7.7092	0.203	-0.187	C	-7.8999	0.5524	-0.7276
H	-7.9384	-0.955	2.5476	H	-8.1584	-0.7757	3.0112	H	-7.5969	-0.4035	2.4958
H	-10.3525	-0.5149	2.8798	H	-10.5798	-0.8243	2.8627	H	-9.9753	-0.755	2.8204
H	-10.6754	-1.1702	0.6137	H	-10.6948	-1.2037	0.4975	H	-10.4926	-1.2051	0.5233
H	-13.311	3.4781	1.7115	H	-12.951	3.8141	1.2309	H	-13.1252	3.5554	1.7124
H	-14.1467	3.9558	0.252	H	-13.3408	4.4378	-0.3568	H	-13.9284	4.0745	0.2488
H	-14.0628	2.2561	0.6999	H	-13.6147	2.7378	0.0131	H	-13.9201	2.3706	0.6891
H	-11.8895	5.1745	-0.6327	H	-9.4779	4.1543	-0.399	H	-11.6126	5.2016	-0.6089
H	-11.0861	4.6989	0.8454	H	-10.426	4.6814	0.9808	H	-10.8427	4.6825	0.8725
H	-10.4015	4.235	-0.703	H	-10.7838	5.3157	-0.607	H	-10.1663	4.1987	-0.6729
H	-12.7699	3.0354	-4.5116	H	-13.2741	2.5816	-3.9245	H	-11.5862	3.1891	-4.1788
H	-12.1703	2.5798	-6.8735	H	-14.0644	0.2889	-3.3687	H	-12.937	3.4034	-6.2454
H	-13.329	1.4157	-6.2574	H	-13.5281	-0.2911	-4.9443	H	-13.9572	2.9638	-4.8885
H	-11.6417	0.9578	-6.4388	H	-14.831	0.8848	-4.8294	H	-13.506	1.7458	-6.0783
H	-10.5481	3.7052	-5.3649	H	-12.2468	1.4195	-6.5502	H	-10.9577	0.9509	-6.1543
H	-10.5661	3.4876	-3.6547	H	-13.6414	2.4438	-6.3708	H	-10.5586	2.6297	-6.3646
H	-8.4837	2.5488	-4.6441	H	-11.7412	3.8096	-7.1264	H	-9.0789	2.5529	-4.3133
H	-9.4039	1.2543	-3.8966	H	-12.1813	4.3101	-5.496	H	-9.4082	0.8286	-4.1627
H	-9.4154	1.4478	-5.6476	H	-10.7633	3.3012	-5.7576	H	-8.5619	1.4394	-5.5743
H	-11.3192	-0.6562	-2.8635	H	-10.6202	-0.5021	-2.9879	H	-11.3533	-0.6648	-2.846
H	-8.6557	2.7463	1.3105	H	-9.3045	2.9767	3.5521	H	-8.6793	1.7497	4.1724
H	-8.4643	3.1036	3.0047	H	-9.265	1.5	4.4981	H	-9.0843	3.1886	3.2547
H	-7.2954	1.0795	3.4226	H	-7.1982	1.3637	3.2121	H	-6.895	1.8425	2.5137
H	-6.7347	1.3807	1.7938	H	-8.003	1.9841	1.7846	H	-8.0286	2.3261	1.2681
H	-9.8	1.2912	4.6936	H	-12.6734	0.9806	3.0999	H	-10.9087	0.5932	4.6374
H	-10.5678	2.8234	4.2859	H	-11.6661	0.6311	4.5033	H	-11.5519	2.1929	4.2799
H	-11.4759	1.3258	4.1482	H	-12.0264	2.299	4.0691	H	-12.1991	0.7818	3.4519
H	-8.6085	-2.1752	-0.8705	H	-8.4482	-2.4638	-0.2914	H	-8.2976	-2.1883	-0.6922
H	-7.1626	-2.0722	0.1305	H	-7.1915	-2.2686	0.9272	H	-6.8646	-1.8087	0.2594
H	-8.6825	-2.6146	0.8279	H	-8.8304	-2.6849	1.4088	H	-8.3128	-2.4169	1.0492

H	-6.8408	0.4315	-0.512	H	-7.8081	-0.2054	-1.1934	H	-6.8155	0.6425	-0.6638
H	-8.2175	0.2111	-1.5812	H	-7.9829	1.2546	-0.2574	H	-8.1373	0.1116	-1.6964
H	-8.2126	1.5285	-0.4389	H	-6.6551	0.1533	0.0861	H	-8.3148	1.5591	-0.7408

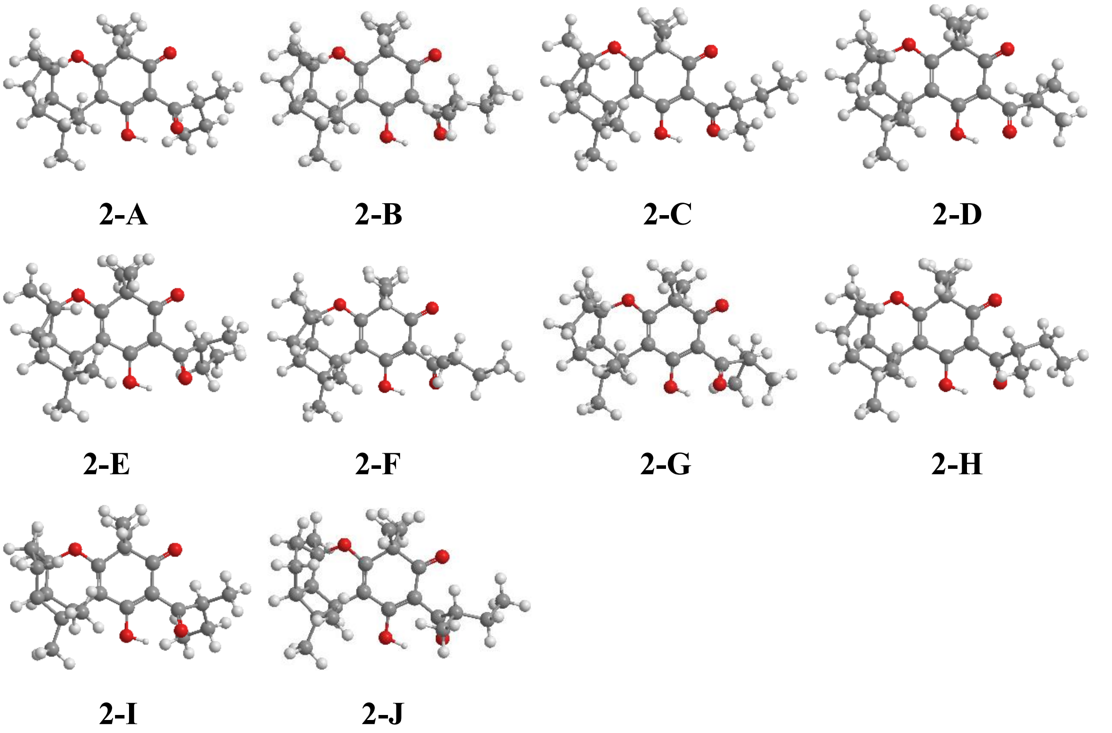

Figure S5. Conformers of isomer 2 for NMR calculation.

Table S5. Important thermodynamic parameters and Boltzmann distributions of the optimized isomer **2** at B3LYP/6-31G* level in the gas phase.

Conformations	Energy (a.u)	ΔG(kcal/mol)	%	Number of imaginary frequencies
2-A	-1196.706294	0	21.47%	0
2-B	-1196.706287	0.004078812	21.32%	0
2-C	-1196.705759	0.335592081	12.18%	0
2-D	-1196.705758	0.335968586	12.17%	0
2-E	-1196.705247	0.656751443	7.08%	0
2-F	-1196.705245	0.657818209	7.07%	0
2-G	-1196.704507	1.12092022	3.23%	0
2-H	-1196.704502	1.124183269	3.22%	0
2-I	-1196.704435	1.166351908	2.99%	0
2-J	-1196.704434	1.166916666	2.99%	0

Table S6. Optimized Z-matrixes of isomer **2** in the gas phase (Å) at B3LYP/6-31G* level.

**2**-A				**2**-B			
C	-1.9856	5.9803	-0.1638	C	-2.0825	5.679	-0.1887
C	-1.4892	6.3813	1.2417	C	-1.4884	5.9644	1.2073

C	-2.5657	6.6973	2.2913	C	-2.4534	6.5057	2.2729
C	-3.9402	6.9907	1.678	C	-3.7391	7.0935	1.6799
C	-4.123	6.9357	0.338	C	-3.9523	7.0784	0.3432
O	-3.203	6.5763	-0.6033	O	-3.1539	6.5183	-0.611
C	-5.0517	7.389	2.543	C	-4.7318	7.7078	2.5626
C	-6.2922	7.714	2.0766	C	-5.8933	8.2655	2.116
C	-6.6045	7.6155	0.6398	C	-6.1805	8.3587	0.6746
C	-5.4567	7.2549	-0.3493	C	-5.191	7.6896	-0.3249
C	-5.2504	8.4899	-1.2696	C	-4.7142	8.7985	-1.3067
C	-5.9146	5.9898	-1.1327	C	-5.9763	6.5525	-1.0372
O	-7.7469	7.8041	0.214	O	-7.1715	8.9597	0.2517
C	-7.3188	8.1782	3.034	C	-6.8607	8.7817	3.1031
O	-7.4233	7.6697	4.1535	O	-6.4839	9.4514	4.068
C	-8.1874	9.4181	2.7106	C	-8.3515	8.3689	3.0205
C	-9.6864	9.0461	2.6597	C	-8.6069	7.1088	3.8767
C	-7.9417	10.5968	3.6889	C	-9.3344	9.508	3.4028
C	-6.5007	11.1413	3.6856	C	-9.2607	10.7411	2.4838
O	-4.7835	7.4388	3.8764	O	-4.437	7.7061	3.8916
C	-2.1802	4.456	-0.1355	C	-2.5955	4.2306	-0.1533
C	-1.2595	3.9416	0.9765	C	-1.7897	3.5321	0.9475
C	-1.119	5.0906	1.9943	C	-1.3925	4.6248	1.9592
C	-0.9952	6.413	-1.2568	C	-1.0416	5.8933	-1.2993
C	-2.2367	5.3759	3.0335	C	-2.4085	5.1434	3.0128
C	-1.6758	5.6137	4.4512	C	-1.7907	5.2523	4.4225
C	-3.3894	4.3467	3.1181	C	-3.7553	4.3877	3.1139
H	-0.1109	5.0725	2.4145	H	-0.4057	4.392	2.3656
H	-0.7022	7.1335	1.1756	H	-0.5591	6.5294	1.1254
H	-2.3021	7.5778	2.8822	H	-1.9946	7.3073	2.8571
H	-4.9175	9.3663	-0.7101	H	-4.1602	9.5872	-0.7938
H	-4.4981	8.2935	-2.0348	H	-4.0571	8.3945	-2.0779
H	-6.166	8.7655	-1.797	H	-5.5481	9.2722	-1.8287
H	-6.8618	6.1475	-1.6526	H	-6.8742	6.924	-1.5356
H	-5.189	5.7067	-1.8962	H	-5.3709	6.0729	-1.8076
H	-6.0425	5.1274	-0.4756	H	-6.2867	5.7713	-0.3407
H	-7.907	9.7669	1.7173	H	-8.5694	8.1012	1.9882
H	-9.8777	8.2569	1.9303	H	-9.6389	6.7697	3.7796
H	-10.05	8.6951	3.6271	H	-7.9682	6.2781	3.5715
H	-10.2966	9.9017	2.3692	H	-8.423	7.2966	4.9362
H	-8.6136	11.4169	3.4297	H	-10.3551	9.1237	3.3693
H	-8.2182	10.303	4.7042	H	-9.1695	9.8161	4.4376
H	-6.4105	12.0066	4.343	H	-10.0131	11.4808	2.7585
H	-5.784	10.396	4.0324	H	-8.2873	11.2296	2.5425
H	-6.1982	11.4544	2.6856	H	-9.4291	10.4679	1.4411

H	-5.6285	7.6035	4.3193	H	-5.1126	8.247	4.324
H	-3.2172	4.2212	0.0983	H	-3.6555	4.2213	0.0946
H	-1.9707	3.9827	-1.0949	H	-2.504	3.7238	-1.1141
H	-0.2781	3.7235	0.5513	H	-0.8835	3.1101	0.509
H	-1.6153	3.0142	1.4271	H	-2.328	2.7018	1.406
H	-1.3249	6.0832	-2.242	H	-1.4499	5.6415	-2.2781
H	-0.0036	5.9969	-1.0802	H	-0.1573	5.277	-1.138
H	-0.9024	7.4986	-1.2908	H	-0.7221	6.9348	-1.3378
H	-2.446	6.0072	5.1161	H	-2.4481	5.8031	5.0968
H	-1.3001	4.6887	4.8895	H	-1.6183	4.2675	4.8575
H	-0.8544	6.3311	4.4483	H	-0.8335	5.7749	4.4073
H	-3.0292	3.3744	3.4542	H	-3.6089	3.3596	3.4447
H	-4.1521	4.6738	3.8254	H	-4.4186	4.8701	3.8324
H	-3.8956	4.2009	2.1671	H	-4.294	4.3581	2.1702

Continued table S6.

2-C				2-D			
C	-2.073	5.6567	-0.2065	C	-1.9398	6.0656	-0.1325
C	-1.4575	5.9273	1.1831	C	-1.4673	6.3422	1.3106
C	-2.4012	6.4784	2.2627	C	-2.5614	6.5782	2.363
C	-3.6855	7.0881	1.6882	C	-3.9226	6.9317	1.7516
C	-3.9144	7.0822	0.3542	C	-4.0805	6.9909	0.4088
O	-3.1361	6.5145	-0.612	O	-3.1434	6.7094	-0.5422
C	-4.6572	7.7151	2.5856	C	-5.0474	7.2663	2.6265
C	-5.8142	8.2984	2.1572	C	-6.2822	7.6226	2.1669
C	-6.1191	8.3932	0.7179	C	-6.5733	7.6186	0.7221
C	-5.1509	7.7151	-0.2961	C	-5.4008	7.3717	-0.273
C	-4.6693	8.8223	-1.2775	C	-5.1853	8.6985	-1.0516
C	-5.9606	6.5932	-1.0057	C	-5.8284	6.1926	-1.1967
O	-7.1109	9.0012	0.3078	O	-7.7155	7.7966	0.2934
C	-6.7613	8.8348	3.1587	C	-7.3184	8.0341	3.1421
O	-6.3584	9.4187	4.1683	O	-7.4417	7.429	4.2106
C	-8.2775	8.5545	3.0322	C	-8.1725	9.3099	2.8935
C	-8.7957	7.7384	4.2413	C	-9.2976	9.4996	3.9445
C	-9.071	9.871	2.8244	C	-7.2781	10.5744	2.7717
C	-10.5356	9.6543	2.4093	C	-8.0138	11.8061	2.2178
O	-4.3456	7.6967	3.9103	O	-4.7963	7.2266	3.9636
C	-2.608	4.2164	-0.1703	C	-2.1479	4.5463	-0.2359
C	-1.7991	3.5009	0.9172	C	-1.2525	3.9312	0.8452
C	-1.3726	4.5831	1.9283	C	-1.1211	4.9894	1.9582
C	-1.0425	5.8592	-1.3289	C	-0.925	6.5784	-1.1668
C	-2.3673	5.1125	2.9966	C	-2.2551	5.1971	2.9983
C	-1.7302	5.2064	4.3988	C	-1.7179	5.3118	4.4403

C	-3.7237	4.3764	3.1118	C	-3.4166	4.1746	2.9772
H	-0.3844	4.3336	2.3213	H	-0.1209	4.9264	2.3928
H	-0.5208	6.4786	1.0921	H	-0.6737	7.0902	1.3227
H	-1.9233	7.2706	2.8444	H	-2.3031	7.4044	3.0299
H	-4.0277	8.4126	-2.0587	H	-4.4133	8.5891	-1.8146
H	-5.5022	9.3112	-1.787	H	-6.0913	9.0214	-1.5686
H	-4.0972	9.5996	-0.7672	H	-4.874	9.5132	-0.3949
H	-6.8587	6.9801	-1.4918	H	-6.7648	6.3971	-1.7198
H	-5.371	6.1093	-1.7855	H	-5.0835	5.9968	-1.969
H	-6.2743	5.8126	-0.3099	H	-5.963	5.2648	-0.6374
H	-8.4141	7.9376	2.1445	H	-8.6712	9.1593	1.9381
H	-9.8315	7.4304	4.1011	H	-9.9667	10.317	3.6775
H	-8.214	6.8272	4.3899	H	-9.9171	8.6049	4.0287
H	-8.747	8.3147	5.1669	H	-8.8932	9.7115	4.9354
H	-9.034	10.4787	3.7311	H	-6.8421	10.8181	3.7428
H	-8.5918	10.4712	2.0469	H	-6.4332	10.3788	2.1082
H	-11.0267	10.6071	2.2093	H	-7.3257	12.6406	2.079
H	-10.6026	9.0541	1.5009	H	-8.4695	11.5916	1.2501
H	-11.1089	9.1518	3.1883	H	-8.801	12.1465	2.8904
H	-5.0115	8.2397	4.3565	H	-5.6476	7.3554	4.4062
H	-3.6647	4.2224	0.0912	H	-3.191	4.3029	-0.0411
H	-2.5368	3.7123	-1.1343	H	-1.9247	4.153	-1.2279
H	-0.905	3.067	0.4655	H	-0.2652	3.7398	0.4206
H	-2.3443	2.6769	1.3791	H	-1.6244	2.9729	1.2097
H	-1.4668	5.6179	-2.3036	H	-1.2387	6.3358	-2.1822
H	-0.7071	6.8957	-1.3674	H	-0.8219	7.6619	-1.1078
H	-0.1661	5.2285	-1.181	H	0.0595	6.1387	-1.0078
H	-2.3708	5.7642	5.0835	H	-2.4969	5.6559	5.1221
H	-1.5672	4.2174	4.8278	H	-1.3571	4.3504	4.8069
H	-0.7656	5.7147	4.3736	H	-0.8912	6.0196	4.511
H	-3.5883	3.3447	3.436	H	-3.07	3.1751	3.2396
H	-4.3704	4.8651	3.8411	H	-4.1905	4.4501	3.6945
H	-4.2751	4.3593	2.1751	H	-3.9053	4.1111	2.0082

Continued table S6.

2-E				2-F				2-G			
C	-1.9641	5.8988	-0.1988	C	-2.1507	5.5303	-0.2105	C	-1.9645	6.0816	-0.1253
C	-1.4388	6.2571	1.2077	C	-1.4942	5.9035	1.136	C	-1.5078	6.4434	1.304
C	-2.4922	6.5919	2.2747	C	-2.4003	6.5556	2.1911	C	-2.6137	6.6958	2.3402
C	-3.8622	6.9444	1.6825	C	-3.6898	7.139	1.6016	C	-3.9819	6.9775	1.7078
C	-4.061	6.9195	0.3441	C	-3.956	7.0271	0.2792	C	-4.1351	6.9661	0.3632
O	-3.1644	6.5449	-0.6138	O	-3.2151	6.3661	-0.6566	O	-3.1866	6.6643	-0.57
C	-4.9497	7.3662	2.567	C	-4.6286	7.8513	2.4696	C	-5.1209	7.3176	2.5617

C	-6.1843	7.7426	2.1223	C	-5.7827	8.4252	2.0223	C	-6.3609	7.6241	2.0809
C	-6.5138	7.6786	0.6862	C	-6.1139	8.4268	0.5869	C	-6.6408	7.5642	0.6354
C	-5.3897	7.297	-0.322	C	-5.1999	7.6273	-0.3884	C	-5.4621	7.2761	-0.3409
C	-5.1502	8.5408	-1.2223	C	-4.727	8.6196	-1.4904	C	-5.2708	8.5534	-1.2049
C	-5.8993	6.063	-1.1231	C	-6.0619	6.4646	-0.9549	C	-5.8666	6.032	-1.1853
O	-7.6528	7.9112	0.2746	O	-7.0827	9.0515	0.1487	O	-7.7787	7.7329	0.1909
C	-7.1862	8.2228	3.1016	C	-6.7057	9.0333	3.0052	C	-7.4169	8.0357	3.0344
O	-7.2705	7.72	4.2256	O	-6.2669	9.7242	3.9287	O	-7.5314	7.4624	4.1213
C	-8.0544	9.4671	2.796	C	-8.2221	8.6964	2.9667	C	-8.3079	9.2759	2.7379
C	-9.5506	9.0832	2.6995	C	-8.4526	7.181	3.19	C	-9.3579	9.5632	3.8414
C	-7.8039	10.5975	3.8294	C	-9.0651	9.563	3.9444	C	-7.4936	10.554	2.3885
C	-8.4069	11.9532	3.4249	C	-10.5845	9.4538	3.7309	C	-6.5084	11.0372	3.4713
O	-4.6628	7.3822	3.8973	O	-4.2916	7.9214	3.7865	O	-4.8794	7.3332	3.9011
C	-2.2119	4.382	-0.195	C	-2.701	4.105	-0.0441	C	-2.1225	4.5531	-0.1553
C	-1.298	3.8159	0.8974	C	-1.8729	3.4688	1.0776	C	-1.2132	4.0209	0.9577
C	-1.1062	4.941	1.9334	C	-1.4073	4.6234	1.9862	C	-1.122	5.1356	2.0183
C	-0.971	6.3162	-1.2953	C	-1.1491	5.6279	-1.3724	C	-0.9621	6.5767	-1.1803
C	-2.202	5.2467	2.99	C	-2.3677	5.2525	3.0316	C	-2.2679	5.357	3.0424
C	-1.6179	5.4394	4.4052	C	-1.6947	5.448	4.4063	C	-1.7428	5.5573	4.4795
C	-3.3893	4.2572	3.0696	C	-3.7311	4.5498	3.2377	C	-3.3965	4.2983	3.0656
H	-0.0949	4.8801	2.3417	H	-0.4127	4.3924	2.3741	H	-0.1228	5.1262	2.4595
H	-0.6266	6.9823	1.1457	H	-0.5528	6.4306	0.9758	H	-0.7383	7.216	1.2819
H	-2.191	7.4519	2.8779	H	-1.8954	7.3836	2.6949	H	-2.3844	7.5607	2.9675
H	-4.4141	8.3323	-1.9999	H	-4.1184	9.427	-1.0786	H	-4.4965	8.4095	-1.9598
H	-6.0614	8.8582	-1.7337	H	-4.1244	8.1184	-2.249	H	-6.182	8.8252	-1.7419
H	-4.7801	9.3945	-0.6512	H	-5.5654	9.0795	-2.0176	H	-4.9753	9.4147	-0.6027
H	-6.8455	6.2629	-1.63	H	-5.5122	5.8893	-1.7011	H	-6.8065	6.1845	-1.7198
H	-5.1918	5.7689	-1.8995	H	-6.3702	5.7661	-0.1746	H	-5.117	5.8007	-1.9432
H	-6.0506	5.1936	-0.4804	H	-6.9666	6.8274	-1.4472	H	-5.9837	5.14	-0.5669
H	-7.7388	9.8453	1.8243	H	-8.5665	8.9448	1.9647	H	-8.8846	9.0146	1.8518
H	-10.1588	9.9228	2.3646	H	-9.5	6.9117	3.0569	H	-9.9958	10.406	3.574
H	-9.7135	8.2759	1.9832	H	-7.8881	6.5739	2.4805	H	-10.0119	8.7038	3.9982
H	-9.945	8.7513	3.6613	H	-8.1592	6.8732	4.1949	H	-8.8901	9.7939	4.7995
H	-8.1946	10.3043	4.8066	H	-8.8222	9.3082	4.9781	H	-6.9456	10.3935	1.4583
H	-6.7298	10.7382	3.97	H	-8.7892	10.6139	3.8273	H	-8.1887	11.3629	2.1566
H	-8.1453	12.7264	4.148	H	-11.1181	10.1463	4.3828	H	-6.0006	11.9487	3.1544
H	-8.0394	12.2762	2.4501	H	-10.858	9.6946	2.7028	H	-7.0196	11.2588	4.4084
H	-9.4951	11.9131	3.3753	H	-10.9558	8.4532	3.9516	H	-5.7379	10.2943	3.6802
H	-5.4959	7.5683	4.3545	H	-4.9345	8.5126	4.2031	H	-5.7372	7.4591	4.3319
H	-3.254	4.1794	0.0463	H	-3.7506	4.1464	0.2416	H	-3.1581	4.2856	0.0472
H	-2.0294	3.9188	-1.1648	H	-2.6602	3.5236	-0.9654	H	-1.8813	4.1196	-1.1261
H	-0.3295	3.5713	0.4573	H	-0.9961	2.9871	0.6408	H	-0.218	3.8413	0.5467
H	-1.6813	2.8936	1.3357	H	-2.4158	2.6928	1.6186	H	-1.5557	3.0698	1.3673

H	-1.3228	6.0159	-2.2824	H	-0.2761	4.9997	-1.197	H	-1.263	6.2749	-2.1837
H	-0.8404	7.3982	-1.3113	H	-0.8036	6.6534	-1.5035	H	-0.8949	7.6646	-1.1737
H	0.0073	5.8625	-1.1375	H	-1.6025	5.3143	-2.3129	H	0.0354	6.1777	-0.9971
H	-2.3666	5.8479	5.0855	H	-2.3101	6.0688	5.059	H	-2.5361	5.9089	5.1407
H	-1.2704	4.4942	4.8231	H	-1.5349	4.4939	4.9094	H	-1.3542	4.6264	4.8933
H	-0.7718	6.1276	4.4055	H	-0.7239	5.9371	4.3176	H	-0.9391	6.2934	4.5197
H	-3.0599	3.2671	3.3845	H	-3.6022	3.5447	3.6393	H	-3.0199	3.3236	3.3756
H	-4.1324	4.598	3.7912	H	-4.3522	5.1046	3.9415	H	-4.182	4.5821	3.7667
H	-3.9105	4.1463	2.1221	H	-4.3065	4.4679	2.3191	H	-3.8786	4.1742	2.0992

Continued table S6.

2-H				2-I				2-J			
C	-2.0497	5.6651	-0.2034	C	-1.8434	6.2127	-0.1505	C	-1.8859	5.8527	-0.1742
C	-1.4585	5.9828	1.1867	C	-1.4684	6.2733	1.3461	C	-1.4889	5.8553	1.3179
C	-2.4326	6.5186	2.2469	C	-2.5665	6.6055	2.3695	C	-2.478	6.4322	2.3437
C	-3.7325	7.0707	1.6493	C	-3.9159	6.9544	1.7375	C	-3.7243	7.0592	1.7153
C	-3.9475	7.0319	0.3141	C	-4.1119	6.8166	0.408	C	-3.9618	6.9443	0.3902
O	-3.1392	6.476	-0.6343	O	-3.205	6.417	-0.5248	O	-3.1792	6.3318	-0.5398
C	-4.7345	7.6797	2.5258	C	-5.0151	7.4193	2.5862	C	-4.6956	7.7485	2.5668
C	-5.9092	8.2135	2.0783	C	-6.2655	7.6987	2.1172	C	-5.8751	8.2542	2.1063
C	-6.2057	8.2605	0.634	C	-6.5984	7.4868	0.6981	C	-6.2021	8.2186	0.6713
C	-5.1996	7.6064	-0.3587	C	-5.4569	7.0827	-0.2812	C	-5.2197	7.4944	-0.2959
C	-4.7501	8.7176	-1.3506	C	-5.2756	8.2657	-1.2724	C	-4.7752	8.5398	-1.3592
C	-5.9557	6.4433	-1.0614	C	-5.9081	5.7693	-0.9858	C	-6.0012	6.3011	-0.9152
O	-7.2215	8.8088	0.2005	O	-7.7515	7.6185	0.2798	O	-7.2189	8.7583	0.2272
C	-6.8824	8.7396	3.0652	C	-7.2798	8.2331	3.051	C	-6.8193	8.849	3.0707
O	-6.4999	9.3173	4.0866	O	-7.3592	7.8204	4.2113	O	-6.4235	9.6126	3.9549
C	-8.3939	8.4281	2.9215	C	-8.1675	9.4331	2.6403	C	-8.3045	8.409	3.0756
C	-8.9413	7.6585	4.1472	C	-9.6628	9.0431	2.6457	C	-8.5084	7.2225	4.0436
C	-9.2574	9.6764	2.582	C	-7.9198	10.6917	3.513	C	-9.2945	9.5615	3.3938
C	-9.3139	10.783	3.6536	C	-6.4852	11.2479	3.4409	C	-9.2743	10.7118	2.3706
O	-4.4315	7.6961	3.8523	O	-4.7255	7.5774	3.9069	O	-4.3637	7.8694	3.8817
C	-2.5321	4.207	-0.1462	C	-1.5277	4.7634	-0.5392	C	-1.8858	4.3637	-0.54
C	-1.7092	3.5412	0.9622	C	-1.8834	3.9469	0.7048	C	-2.3933	3.6595	0.72
C	-1.3326	4.6563	1.9572	C	-1.4027	4.8291	1.8747	C	-1.7277	4.4393	1.8721
C	-1.016	5.8854	-1.3196	C	-1.0979	7.2456	-1.0071	C	-0.9509	6.6932	-1.0551
C	-2.3566	5.1683	3.006	C	-2.3323	5.2278	3.0479	C	-2.5426	5.0457	3.0417
C	-1.7377	5.3106	4.4123	C	-1.5715	5.3473	4.3855	C	-1.766	5.017	4.3753
C	-3.6867	4.3856	3.1219	C	-3.5881	4.3482	3.2609	C	-3.9574	4.4613	3.2712
H	-0.3401	4.4501	2.3641	H	-0.4006	4.505	2.1643	H	-0.8149	3.915	2.1636
H	-0.5416	6.5662	1.0945	H	-0.5411	6.8304	1.4862	H	-0.4623	6.2033	1.4398
H	-1.9895	7.3379	2.8184	H	-2.2884	7.4481	3.007	H	-2.0164	7.203	2.9655
H	-4.2151	9.5241	-0.8453	H	-4.5315	8.0336	-2.0355	H	-4.2219	9.3675	-0.9113

H	-4.0834	8.3224	-2.1181	H	-6.201	8.5018	-1.8019	H	-4.1275	8.0919	-2.114
H	-5.5953	9.166	-1.8769	H	-4.9448	9.1755	-0.7677	H	-5.6247	8.9675	-1.8958
H	-6.8621	6.7872	-1.5642	H	-6.8626	5.8854	-1.503	H	-5.4022	5.7774	-1.6615
H	-5.338	5.9718	-1.8269	H	-5.1874	5.4507	-1.7399	H	-6.2881	5.5654	-0.1614
H	-6.2472	5.661	-0.3579	H	-6.0177	4.9447	-0.2788	H	-6.9126	6.6253	-1.4217
H	-8.4867	7.7458	2.0771	H	-7.9071	9.701	1.617	H	-8.5516	8.0511	2.0778
H	-9.9964	7.4145	4.0202	H	-10.2873	9.8655	2.2958	H	-9.5366	6.8604	4.0109
H	-8.4089	6.7181	4.2973	H	-9.8568	8.1943	1.9873	H	-7.8648	6.3788	3.788
H	-8.8476	8.2362	5.0682	H	-10.0069	8.7697	3.6447	H	-8.2933	7.5026	5.0765
H	-8.9067	10.1179	1.6472	H	-8.6047	11.4807	3.1974	H	-10.3086	9.1602	3.4293
H	-10.2749	9.3476	2.3655	H	-8.1774	10.4809	4.5537	H	-9.1003	9.96	4.3921
H	-9.9642	11.5978	3.3344	H	-6.3945	12.1662	4.0219	H	-10.0304	11.4608	2.6072
H	-9.7022	10.4087	4.6007	H	-5.7552	10.5413	3.8376	H	-8.3085	11.2184	2.3532
H	-8.33	11.2128	3.8435	H	-6.2013	11.4789	2.4135	H	-9.4725	10.3472	1.3616
H	-5.1225	8.2205	4.2839	H	-5.5644	7.7731	4.3491	H	-5.0319	8.4425	4.2828
H	-3.591	4.1789	0.1048	H	-2.0778	4.4377	-1.4229	H	-2.5004	4.1477	-1.4147
H	-2.4323	3.6886	-1.1	H	-0.4661	4.6558	-0.7659	H	-0.8728	4.032	-0.7713
H	-0.7954	3.1319	0.5275	H	-1.4167	2.9609	0.707	H	-2.143	2.5979	0.7362
H	-2.229	2.7065	1.434	H	-2.9609	3.7891	0.7226	H	-3.4796	3.7308	0.7465
H	-1.4214	5.6115	-2.2937	H	-1.3196	7.1114	-2.066	H	-1.0255	7.7495	-0.7966
H	-0.1188	5.29	-1.1521	H	-1.3942	8.2582	-0.7335	H	-1.2085	6.5926	-2.1095
H	-0.7184	6.9327	-1.3735	H	-0.0184	7.1667	-0.881	H	0.0896	6.392	-0.9368
H	-2.4049	5.8569	5.0805	H	-2.193	5.8172	5.149	H	-2.2664	5.6213	5.1332
H	-1.5433	4.336	4.861	H	-1.2701	4.3683	4.7593	H	-1.6808	4.0016	4.7633
H	-0.7919	5.8531	4.3869	H	-0.6688	5.9513	4.2862	H	-0.7548	5.4103	4.2641
H	-3.5175	3.3656	3.4665	H	-3.3144	3.3207	3.5003	H	-3.9103	3.4021	3.5243
H	-4.3581	4.8637	3.8358	H	-4.194	4.728	4.0843	H	-4.4613	4.9741	4.0911
H	-4.2274	4.3315	2.1804	H	-4.2411	4.3217	2.3899	H	-4.6053	4.5651	2.4022

4. Spectral information of 1

4.1 HR-ESI-MS, IR and UV (CH_3OH) spectrum of 1b

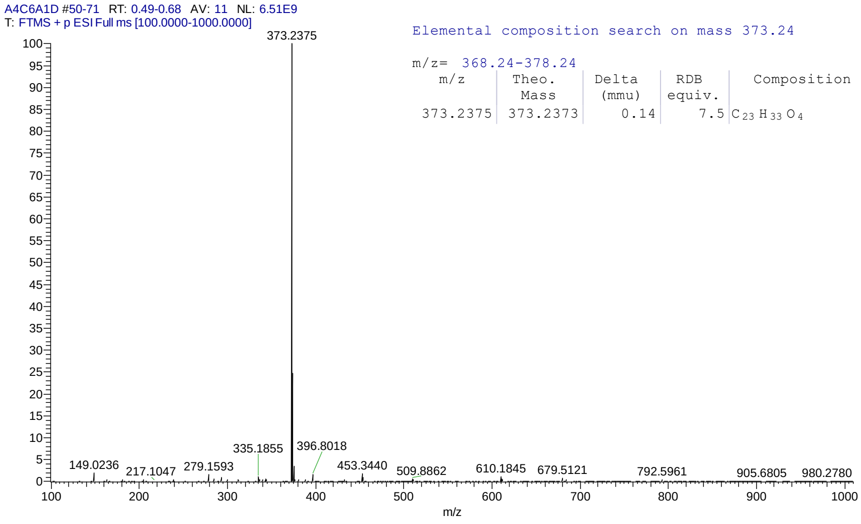

HR-ESI-MS of compound 1b

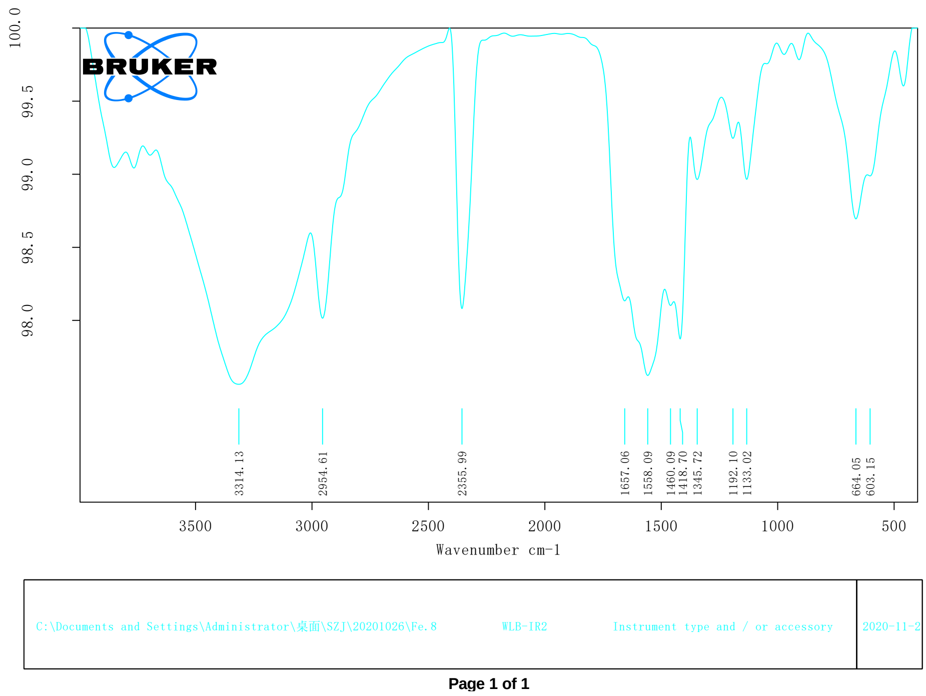

IR spectrum of compound 1b

Spectrum Peak Pick Report

2020-11-02 19:44:46

Data Set: A4C6A1D 0.04mg ml - RawData

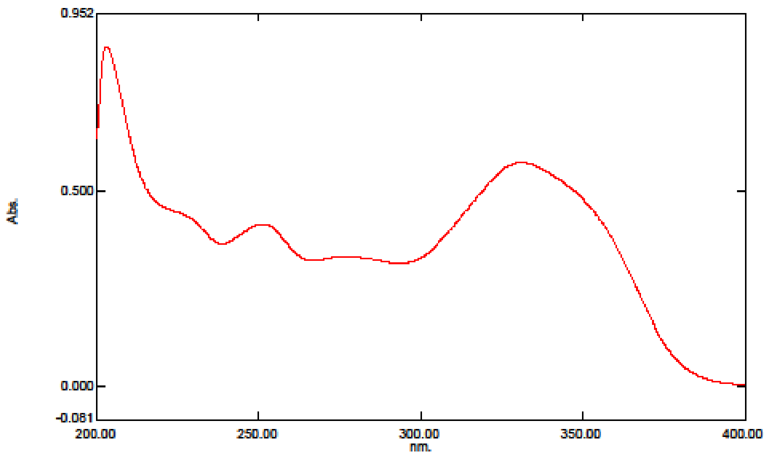

No.	P/V	Wavelength	Abs.	Description
1	P	332.00	0.571	
2	P	276.50	0.332	
3	P	251.00	0.414	
4	P	203.00	0.866	

[Measurement Properties]
Wavelength Range (nm.): 200.00 to 400.00
Scan Speed: Medium
Sampling Interval: 0.5
Auto Sampling Interval: Disabled
Scan Mode: Single

[Instrument Properties]
Instrument Type: UV-2600 Series
Measuring Mode: Absorbance
Slit Width: 2.0
Accumulation time: 0.1 sec.
Light Source Change Wavelength: 323.0 nm
Detector Unit: Direct
S/R Exchange: Normal
Stair Correction: OFF

[Attachment Properties]
Attachment: None

[Operation]
Threshold: 0.0010000
Points: 4
InterPolate: Disabled
Average: Disabled

[Sample Preparation Properties]
Weight:
......

Page 1 / 1

UV spectrum of compound 1b

4.2 1D and 2D NMR spectra of 1 in $CDCl_3$

4.2.1 ^1H NMR spectrum of 1a in $CDCl_3$

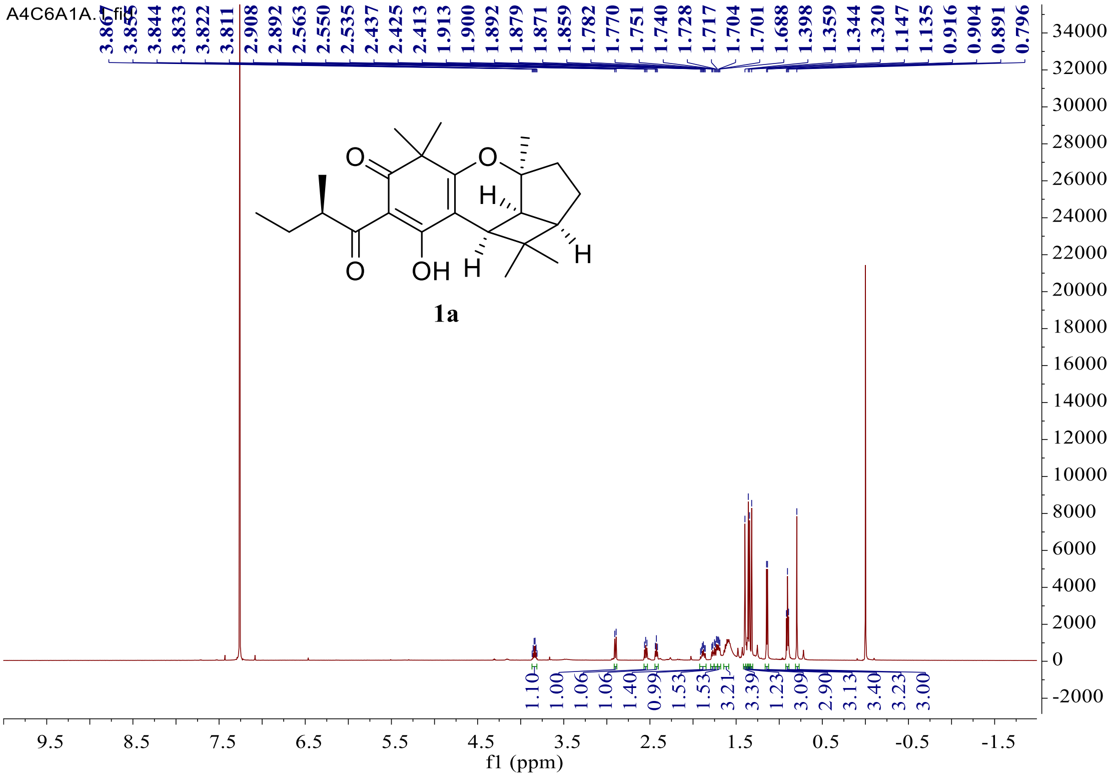

4.2.2 ^1H NMR spectrum of 1b in $CDCl_3$

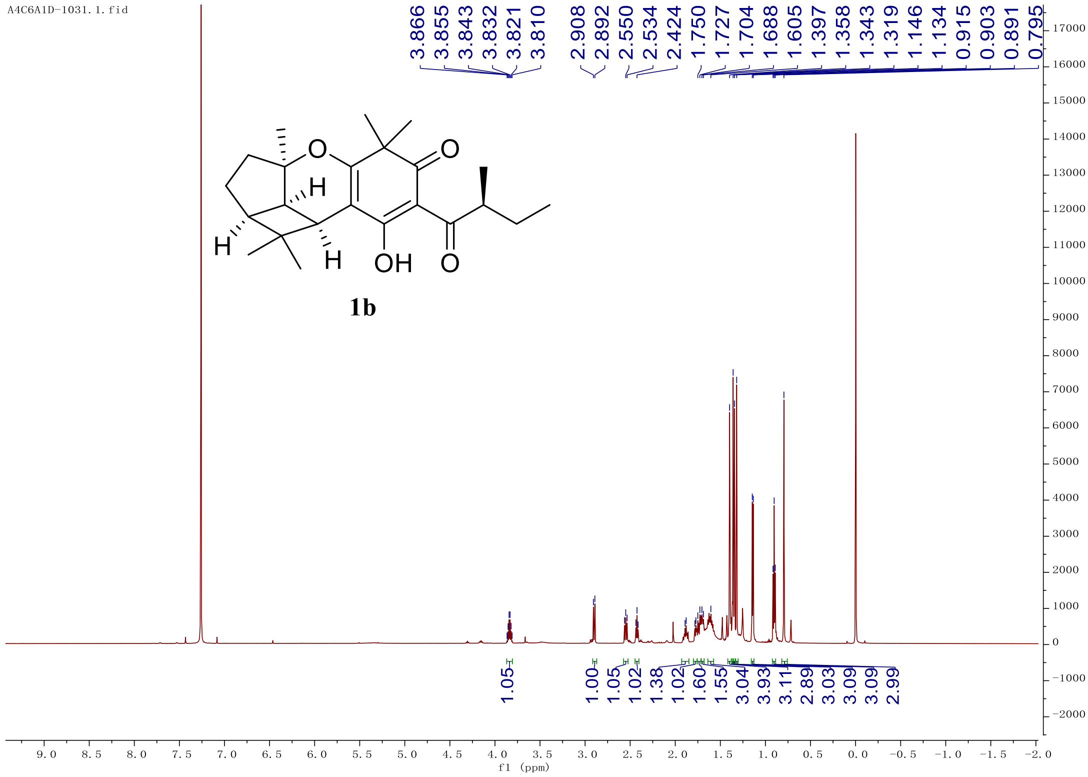

4.2.3 ^{13}C NMR spectrum of 1b in $CDCl_3$

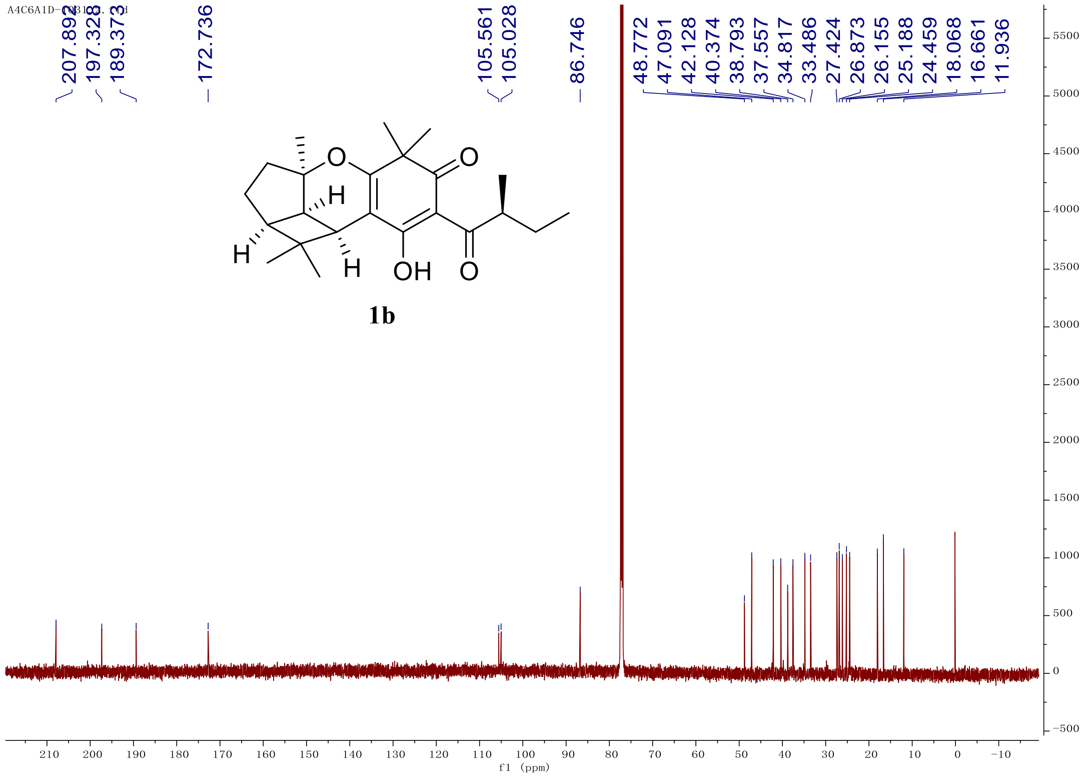

4.2.4 DEPT-135 spectrum of 1b in $CDCl_3$

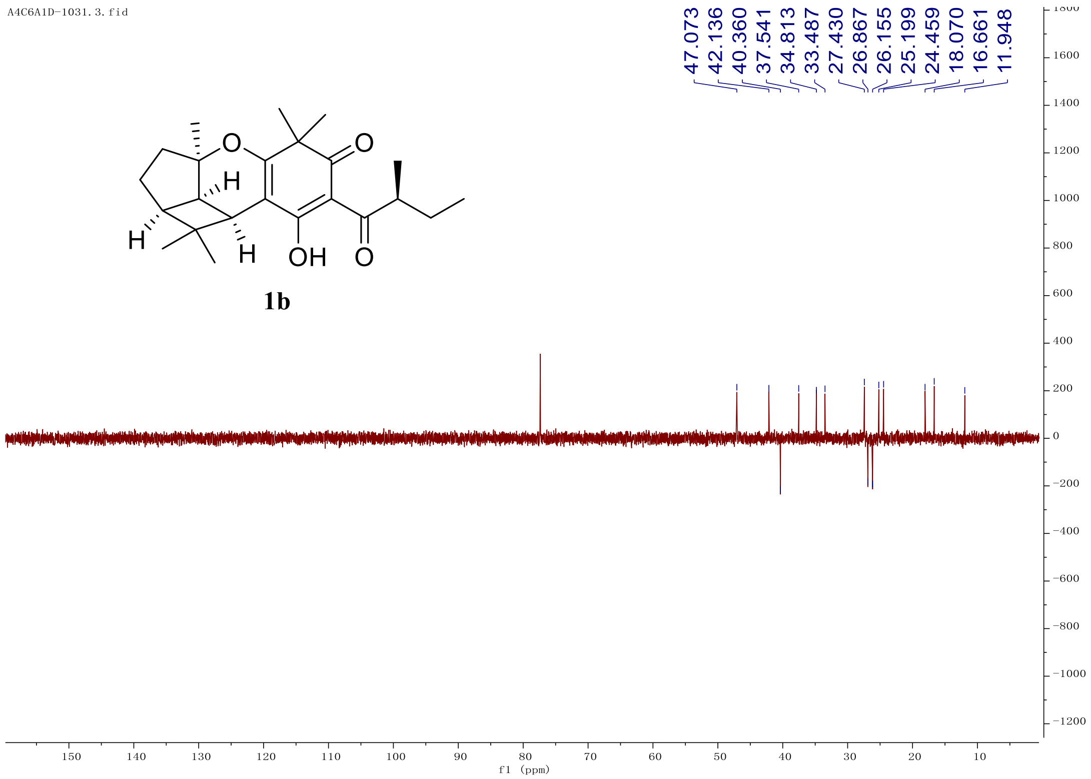

4.2.5 ^1H–^1H COSY spectrum of 1b in $CDCl_3$

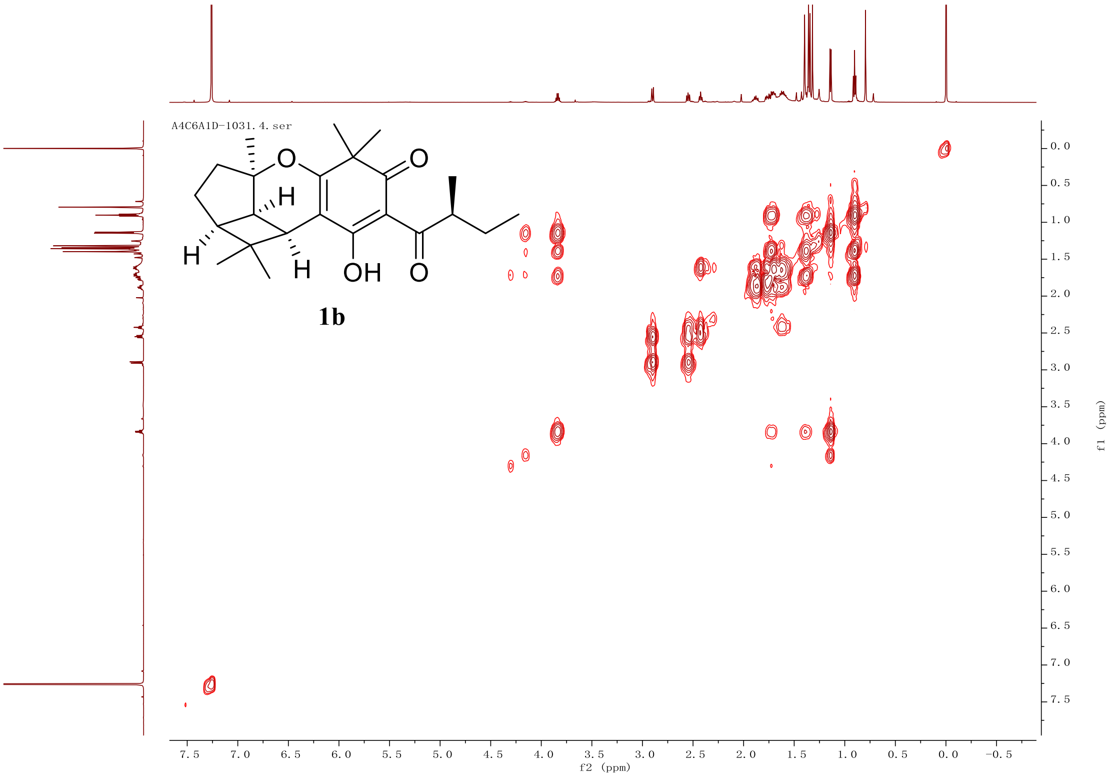

4.2.6 HSQC spectrum of 1b in $CDCl_3$

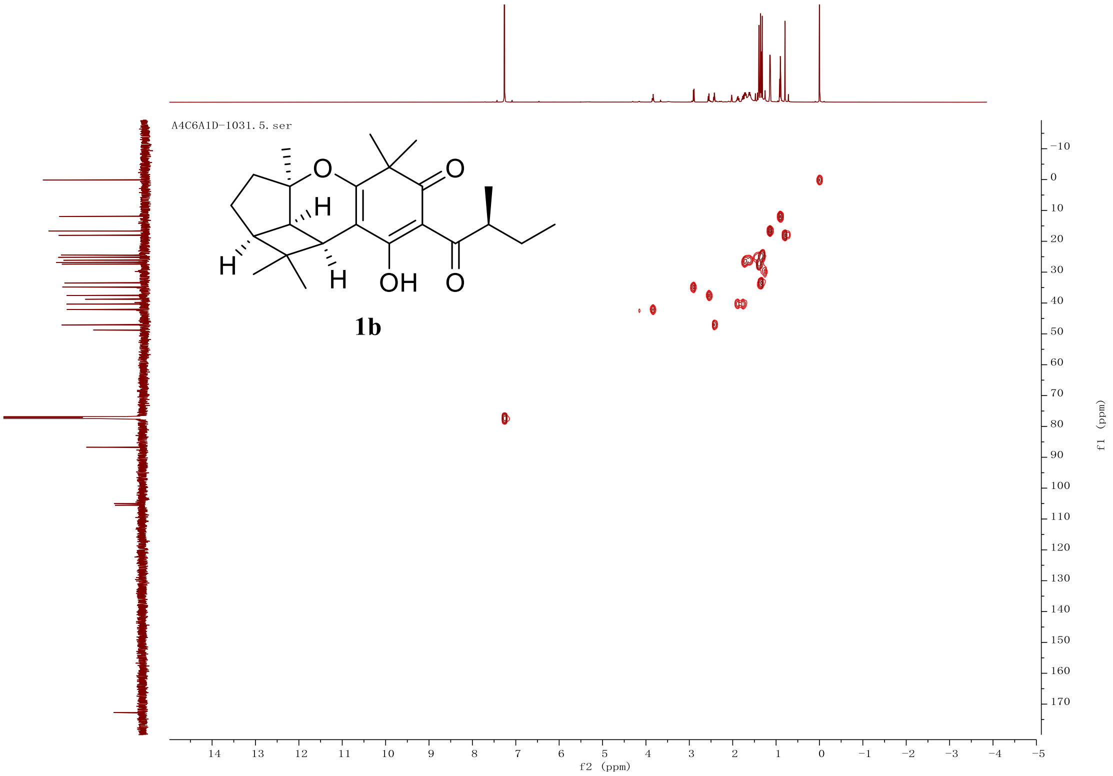

4.2.7 HMBC spectrum of 1b in CDCl$_3$

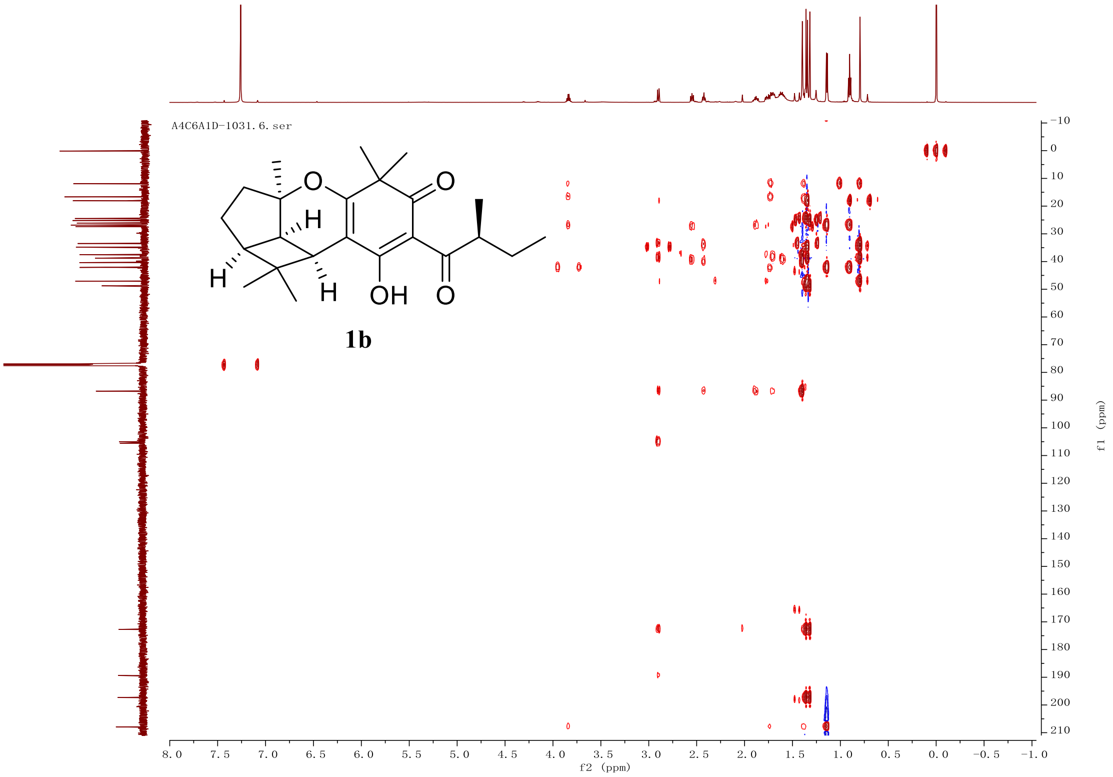

4.2.8 NOESY spectrum of 1b in CDCl$_3$

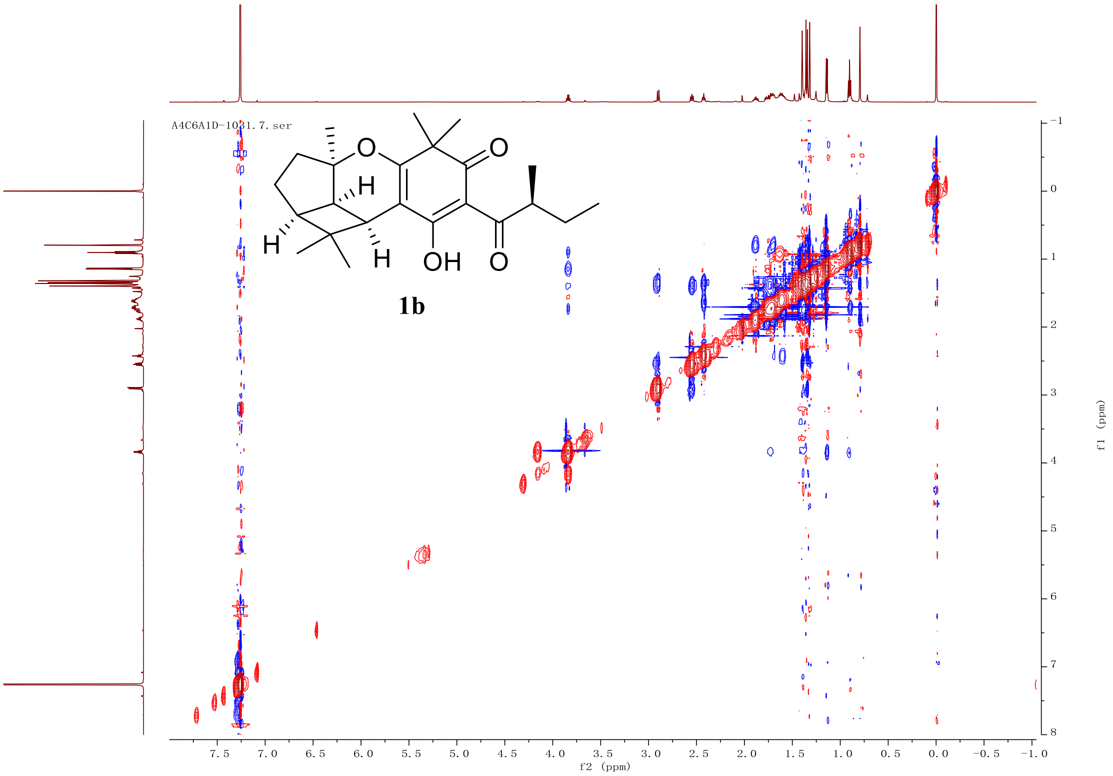

5. Spectral information of 2

5.1 HR-ESI-MS, IR and UV (CH_3OH) spectrum of 2a

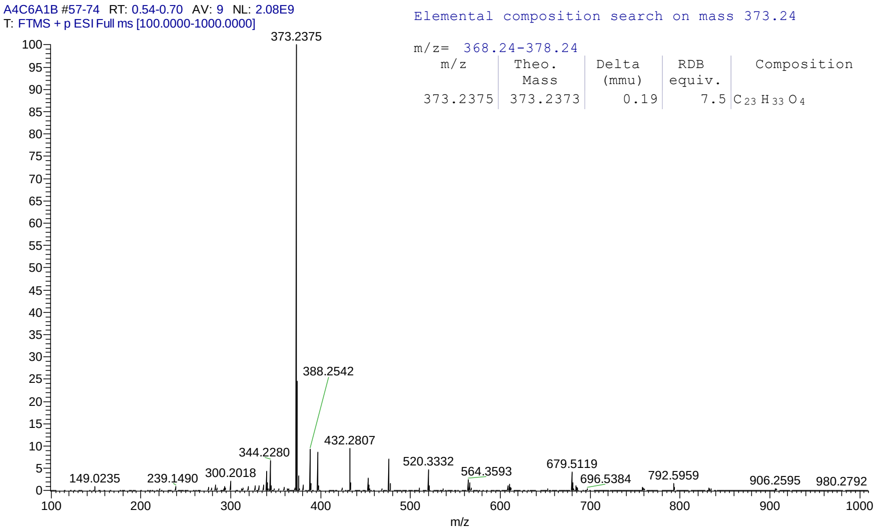

HR-ESI-MS of compound 2a

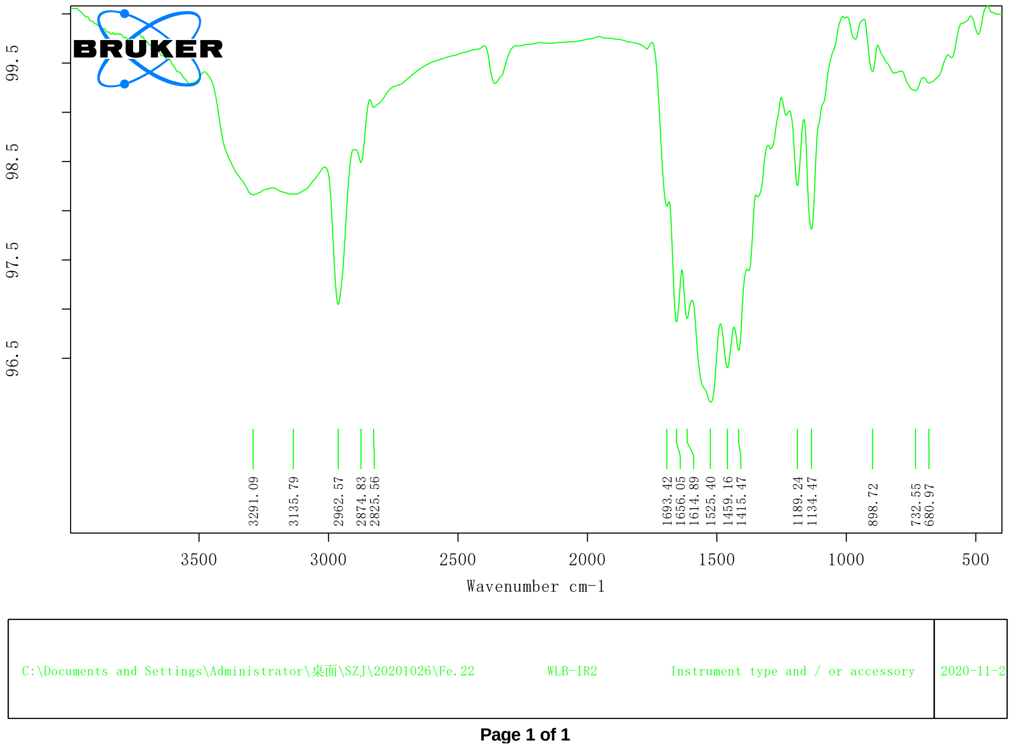

IR spectrum of compound 2a

Spectrum Peak Pick Report

2020-11-02 19:43:19

Data Set: A4C6A1C 0.04mg ml - RawData

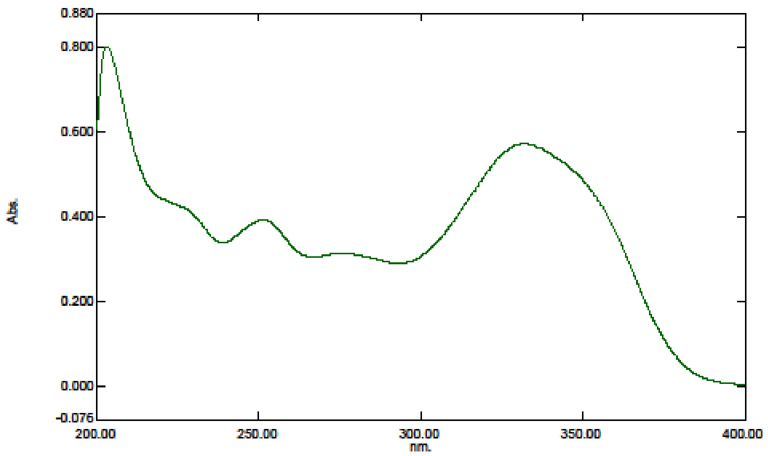

No.	P/V	Wavelength	Abs.	Description
1	⊕	332.00	0.572	
2	⊕	276.00	0.313	
3	⊕	251.50	0.392	
4	⊕	203.00	0.800	

[Measurement Properties]
Wavelength Range (nm.): 200.00 to 400.00
Scan Speed: Medium
Sampling Interval: 0.5
Auto Sampling Interval: Disabled
Scan Mode: Single

[Instrument Properties]
Instrument Type: UV-2600 Series
Measuring Mode: Absorbance
Slit Width: 2.0
Accumulation time: 0.1 sec.
Light Source Change Wavelength: 323.0 nm
Detector Unit: Direct
S/R Exchange: Normal
Stair Correction: OFF

[Attachment Properties]
Attachment: None

[Operation]
Threshold: 0.0010000
Points: 4
InterPolate: Disabled
Average: Disabled

[Sample Preparation Properties]
Weight:
......

Page 1 / 1

UV spectrum of compound 2a

5.2 1D and 2D NMR spectra of 2 in $CDCl_3$

5.2.1 ^{1}H NMR spectrum of 2a in $CDCl_3$

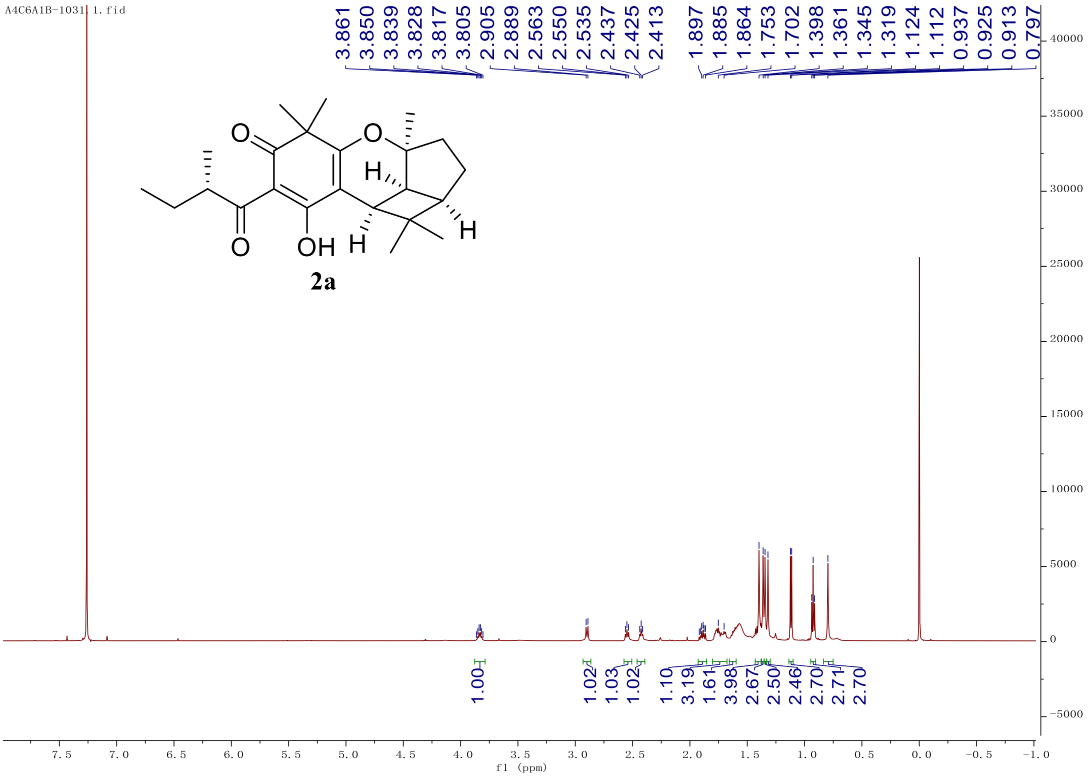

5.2.2 ^{1}H NMR spectrum of 2b in $CDCl_3$

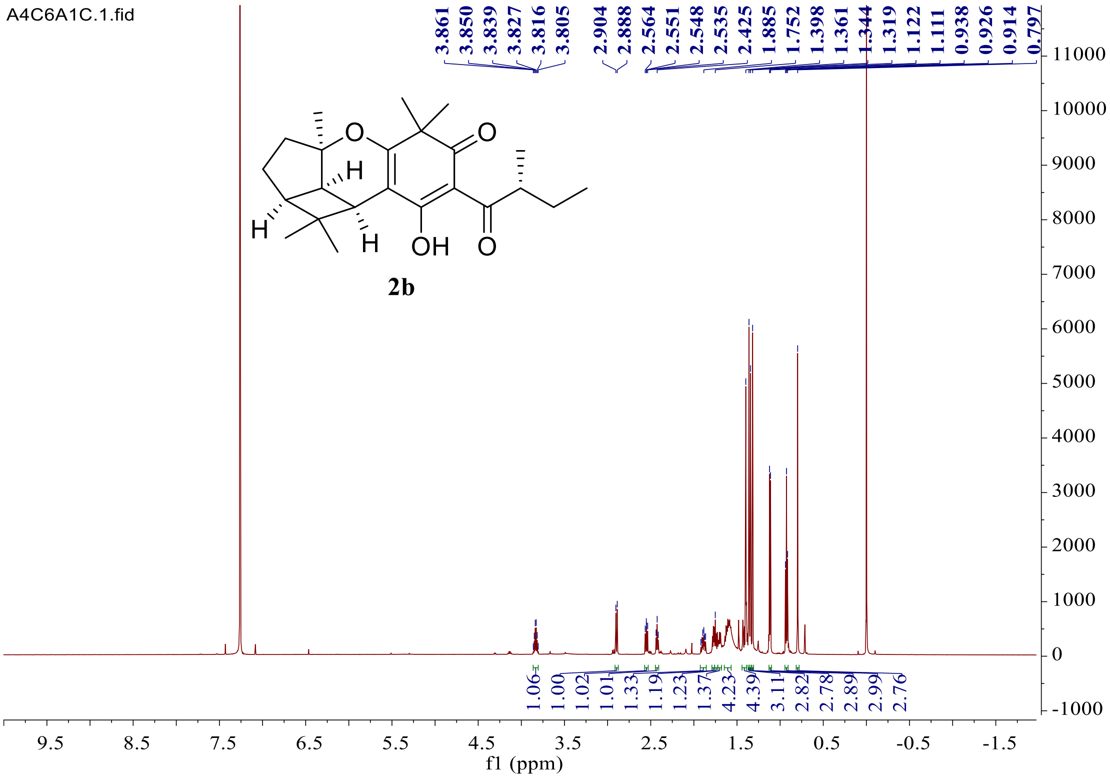

5.2.2 ^{13}C NMR spectrum of 2a in CDCl$_3$

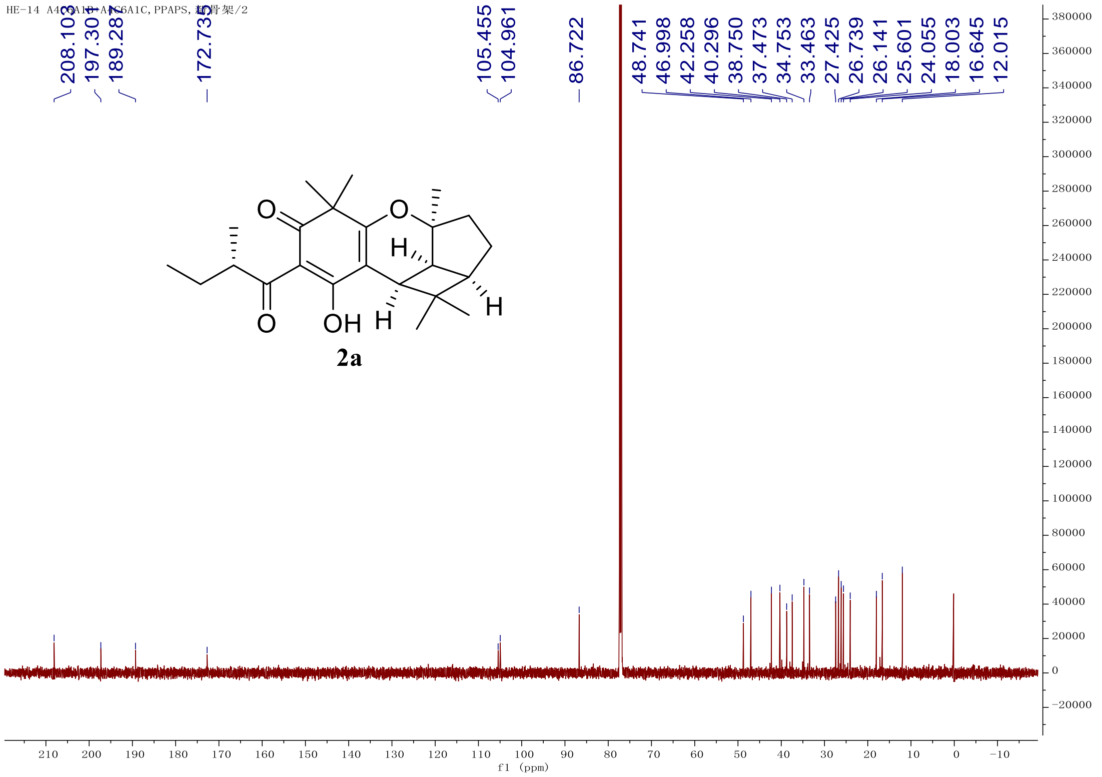

5.2.3 DEPT-135 spectrum of 2a in CDCl$_3$

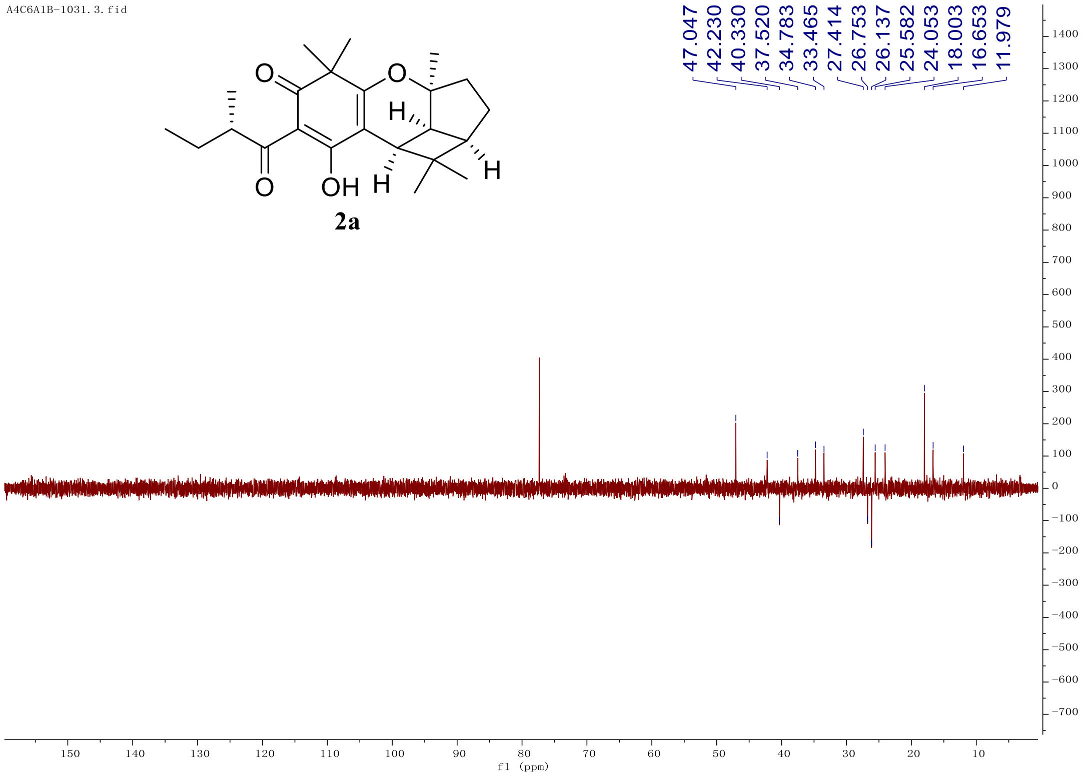

5.2.4 ^{1}H–^{1}H COSY spectrum of 2a in $CDCl_3$

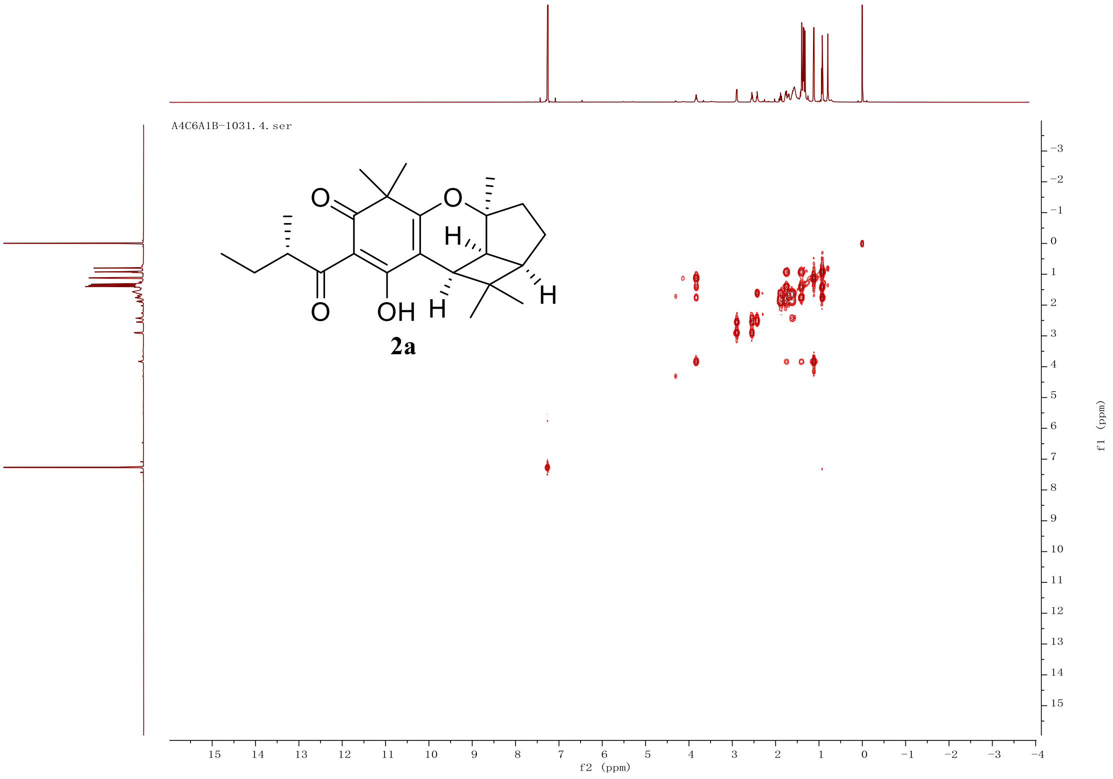

5.2.5 HSQC spectrum of 2a in $CDCl_3$

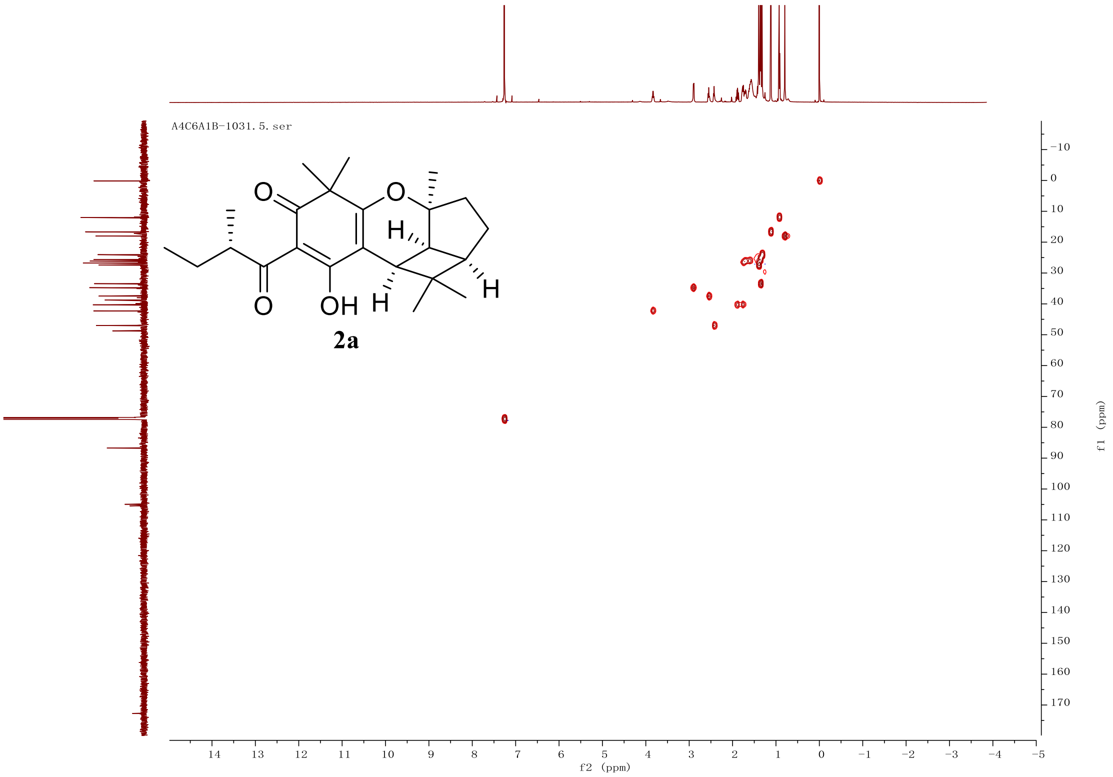

5.2.6 HMBC spectrum of 2a in CDCl$_3$

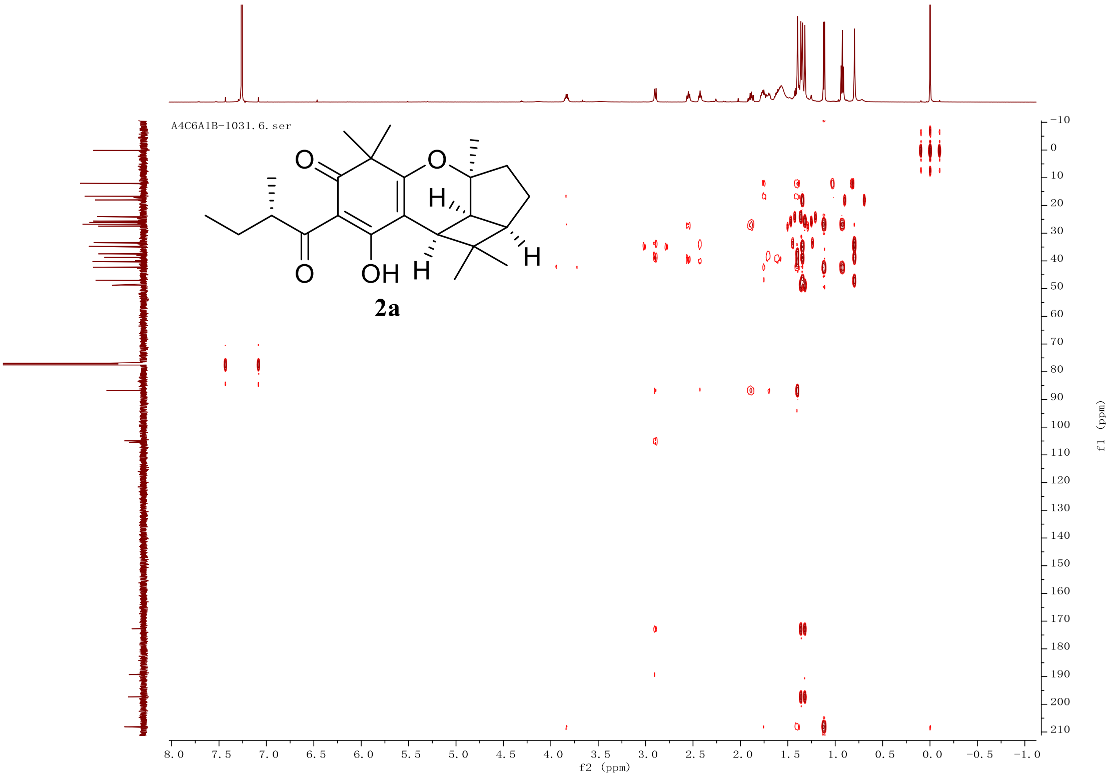

5.2.7 NOESY spectrum of 2a in CDCl$_3$

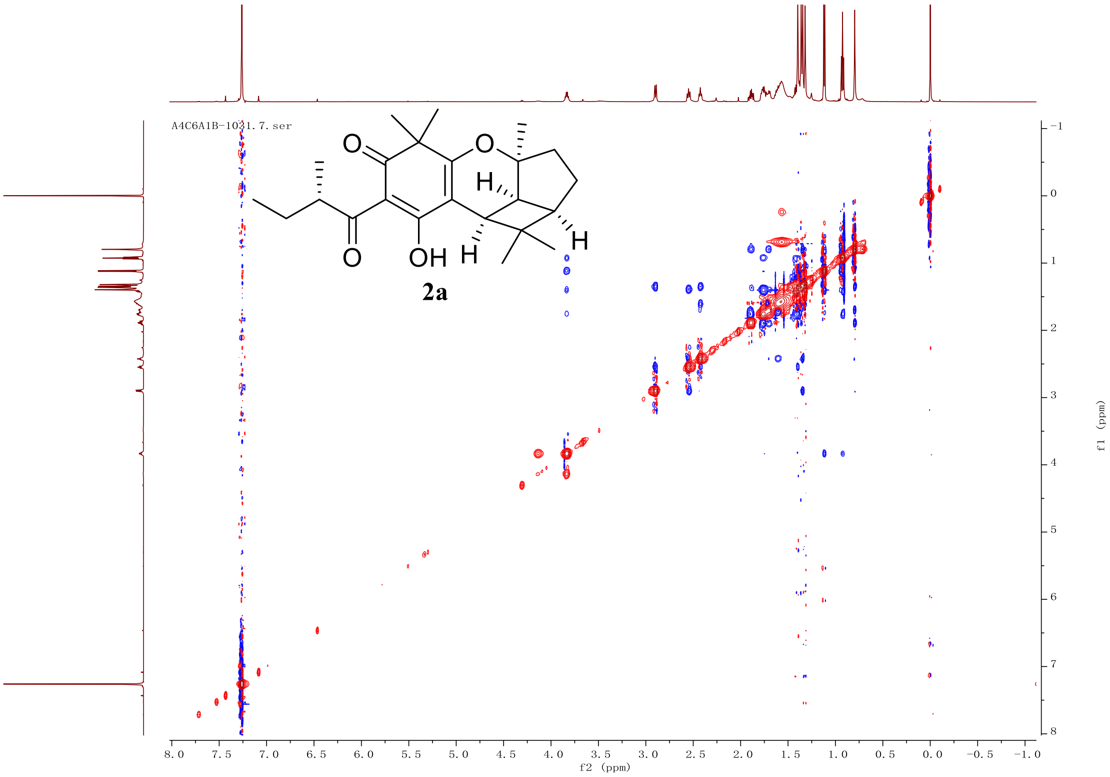

6. Spectral information of 3a

6.1 HR-ESI-MS, IR and UV (CH_3OH) spectrum of 3a

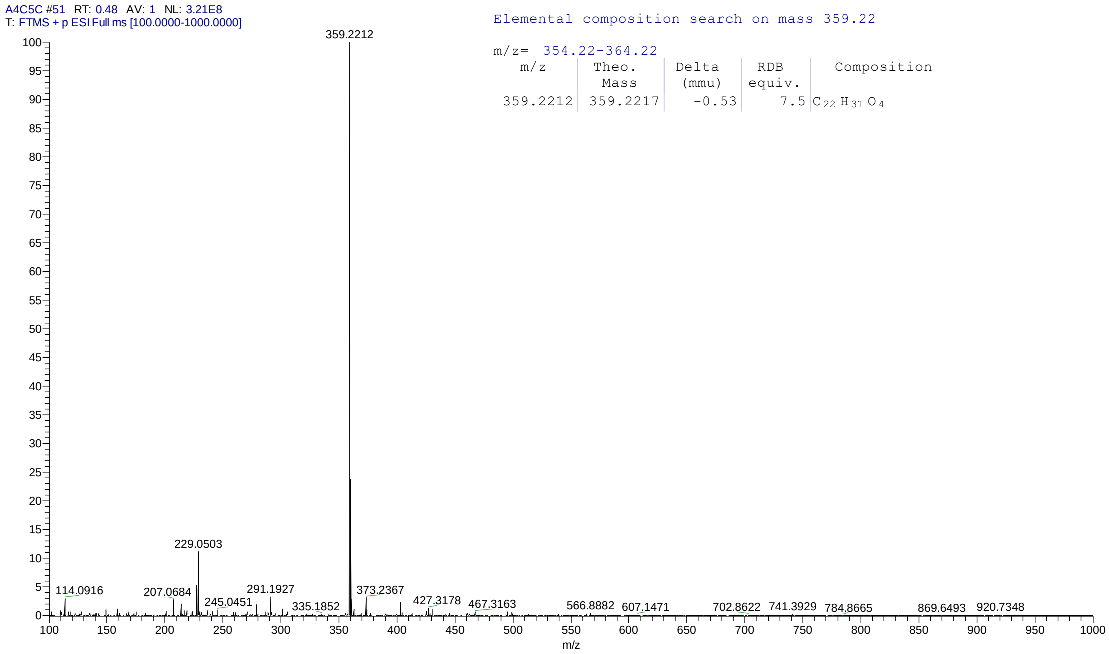

HR-ESI-MS of compound 3a

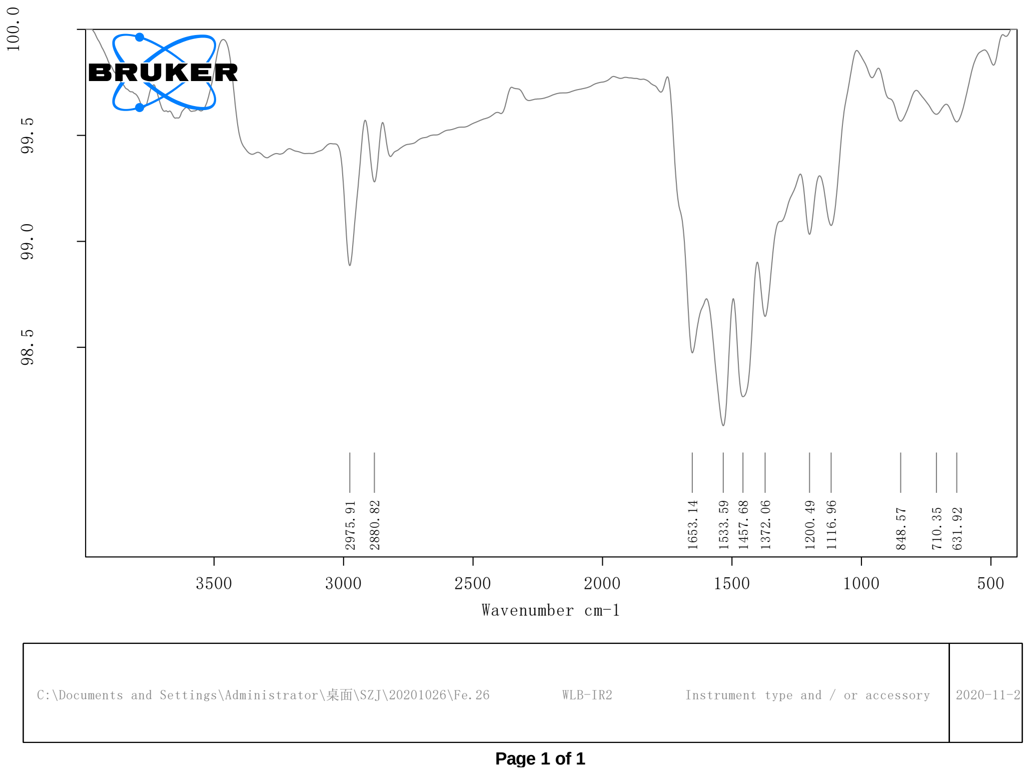

IR spectrum of compound 3a

Spectrum Peak Pick Report

2020-11-17 21:30:56

Data Set: A4C5C2 0.08 - RawData

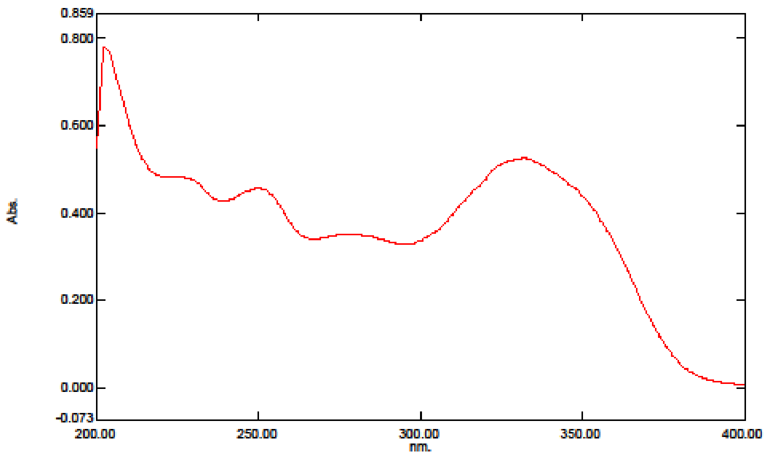

No.	P/V	Wavelength	Abs.	Description
1	P	332.00	0.526	
2	P	278.00	0.350	
3	P	250.00	0.456	

[Measurement Properties]
Wavelength Range (nm.): 200.00 to 400.00
Scan Speed: Medium
Sampling Interval: 2.0
Auto Sampling Interval: Disabled
Scan Mode: Single

[Instrument Properties]
Instrument Type: UV-2600 Series
Measuring Mode: Absorbance
Slit Width: 1.0
Accumulation time: 0.1 sec.
Light Source Change Wavelength: 323.0 nm
Detector Unit: Direct
S/R Exchange: Normal
Stair Correction: OFF

[Attachment Properties]
Attachment: None

[Operation]
Threshold: 0.0010000
Points: 4
InterPolate: Disabled
Average: Disabled

[Sample Preparation Properties]
Weight:
......

Page 1 / 1

UV spectrum of compound 3a

6.2 1D and 2D NMR spectra of 3a in $CDCl_3$

6.2.1 ^{1}H NMR spectrum of 3a in $CDCl_3$

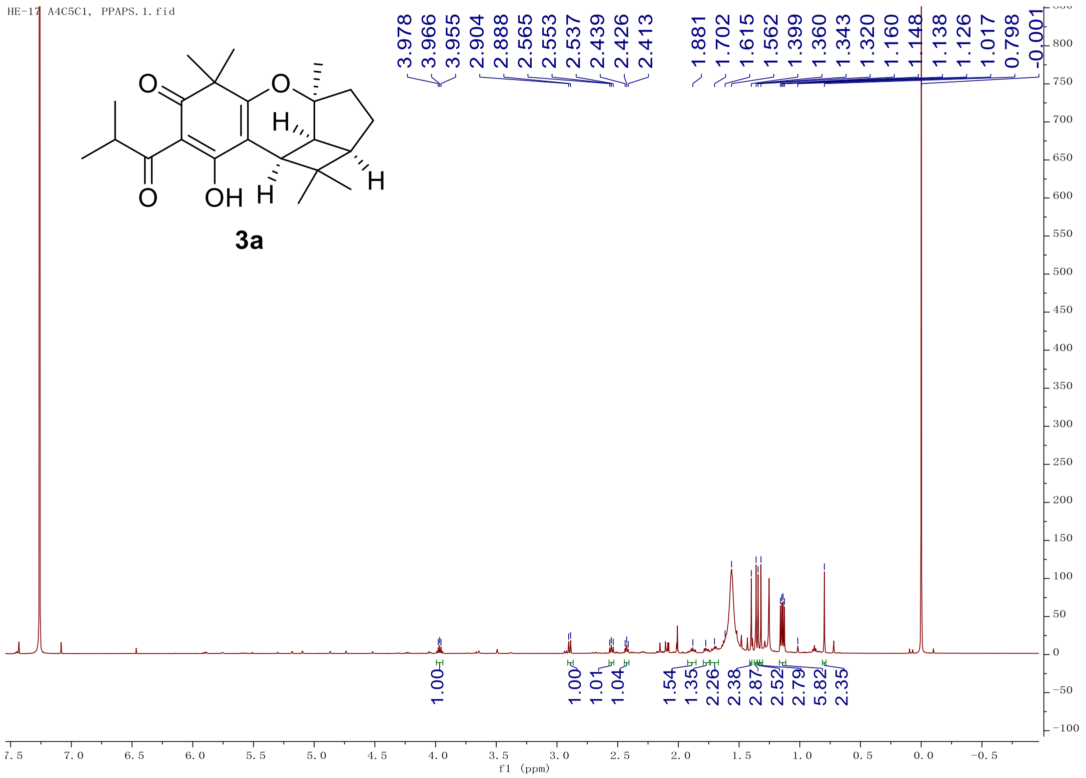

6.2.2 ^{13}C NMR spectrum of 3a in $CDCl_3$

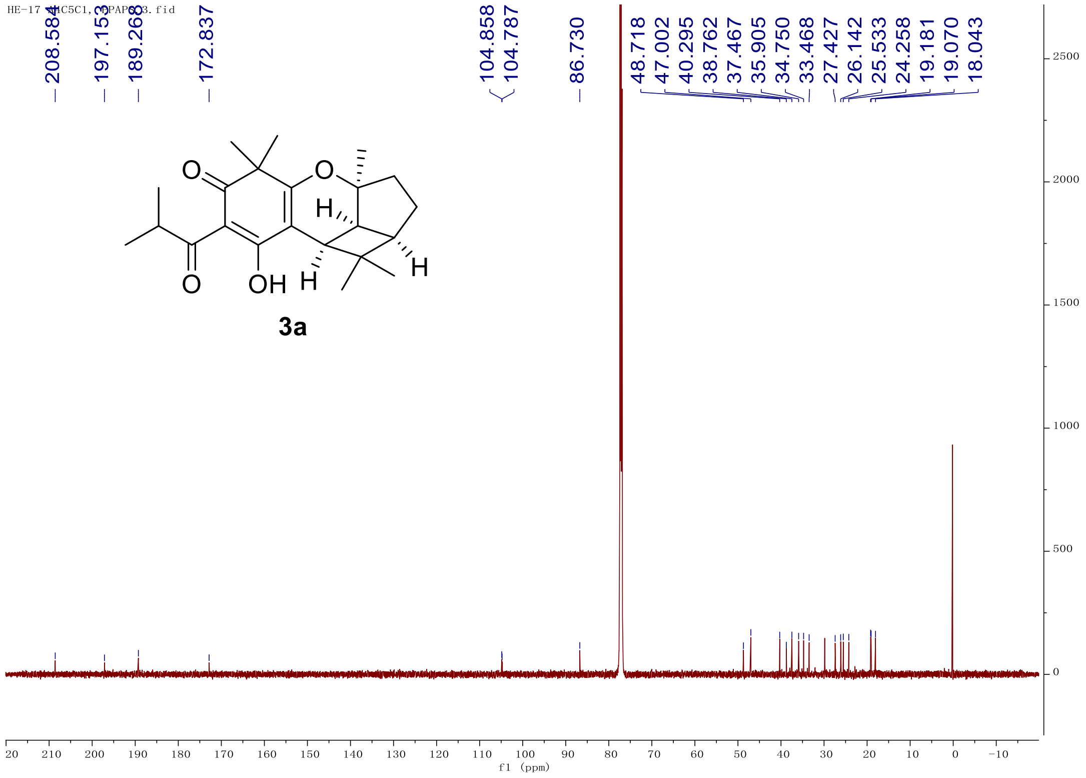

6.2.3 DEPT-135 spectrum of 3a in CDCl$_3$

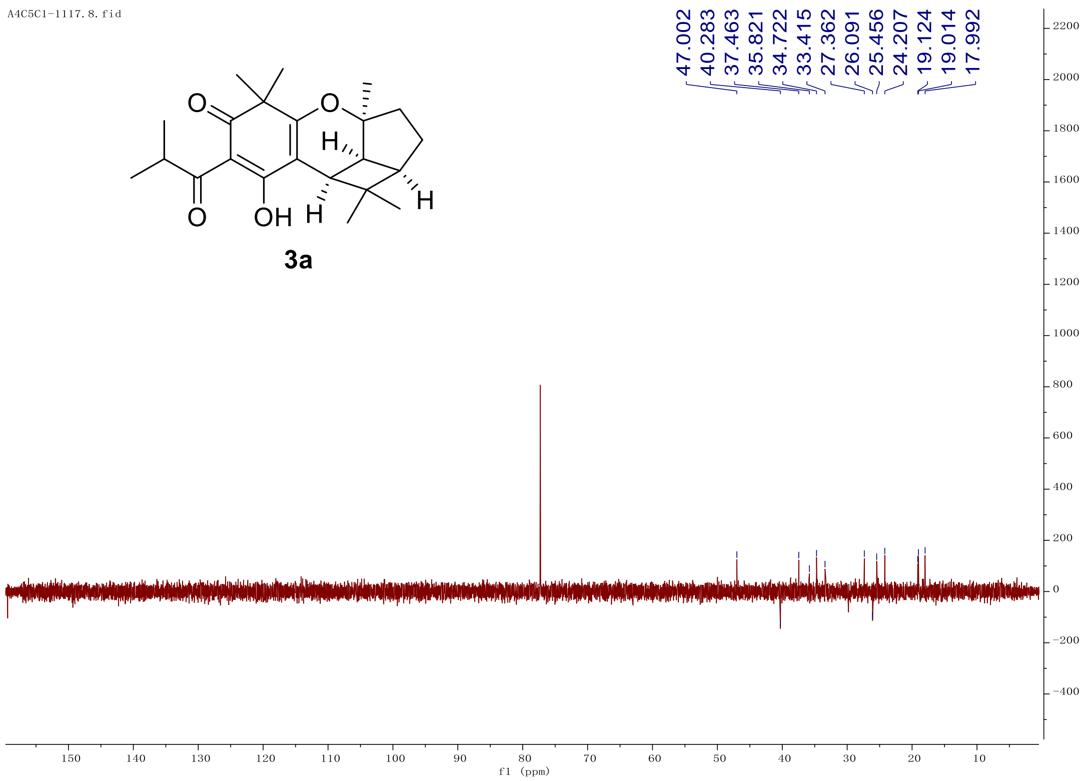

6.2.4 ^{1}H–^{1}H COSY spectrum of 3a in CDCl$_3$

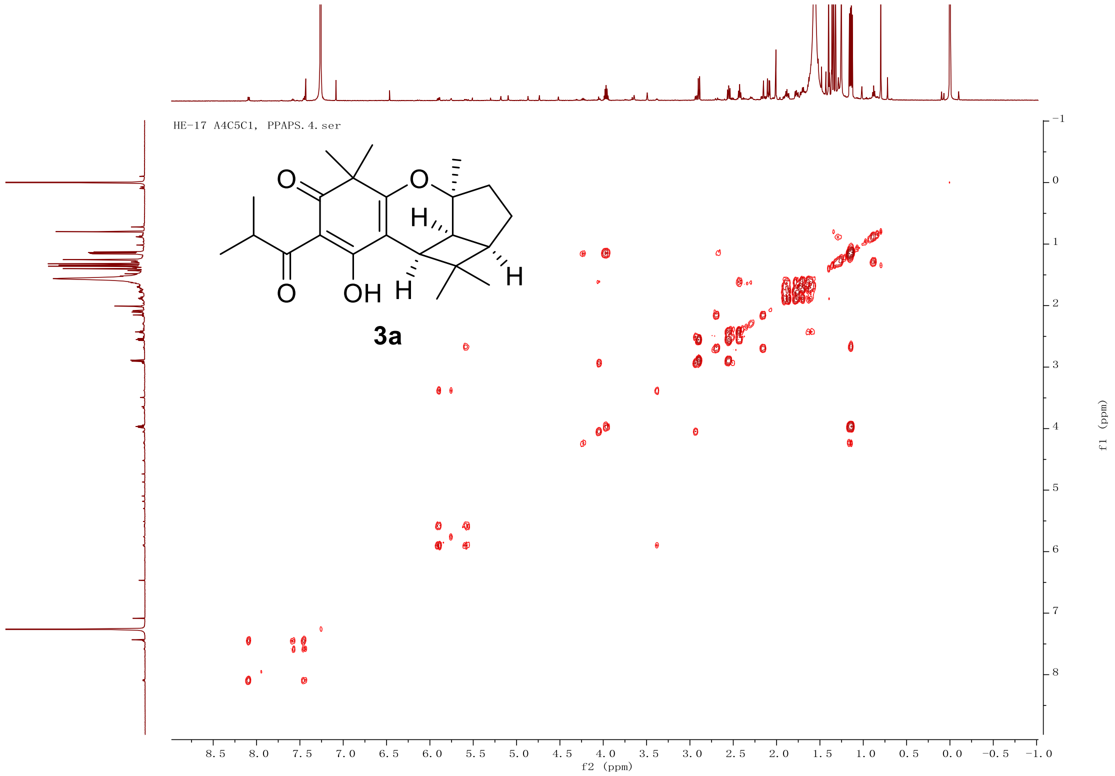

6.2.5 HSQC spectrum of 3a in CDCl$_3$

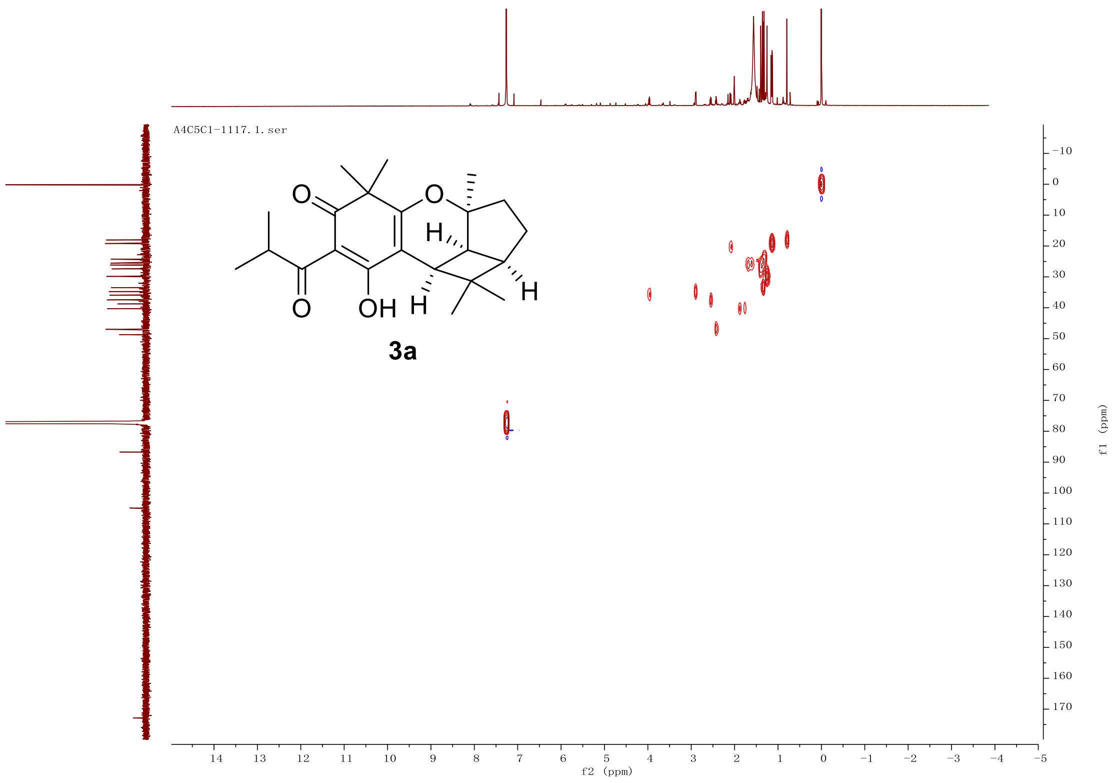

6.2.6 HMBC spectrum of 3a in CDCl$_3$

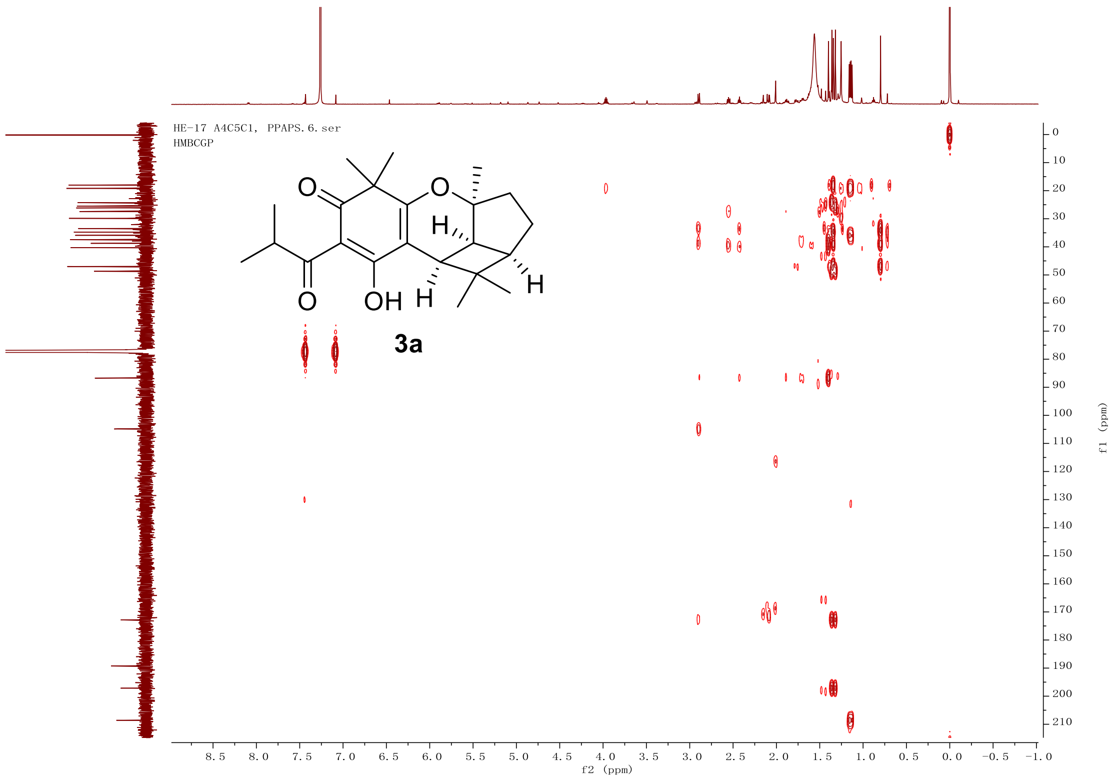

6.2.7 NOESY spectrum of 3a in CDCl3

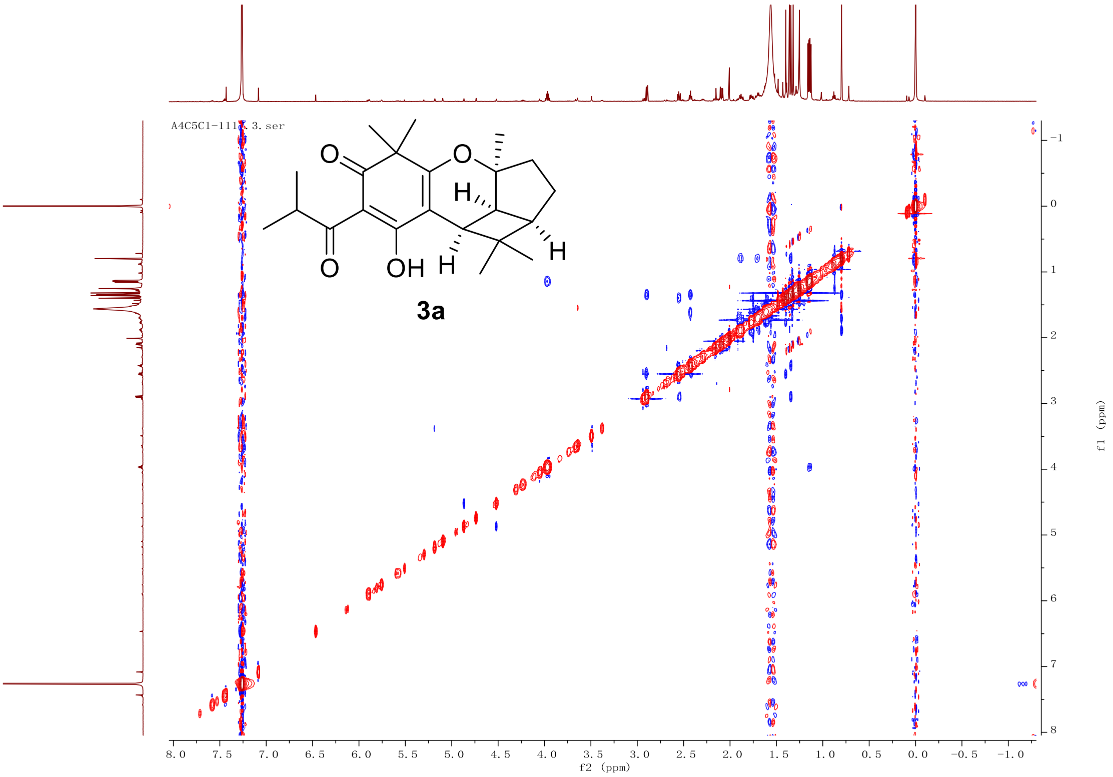

Supplement: Supplementary file 1 [file biomedicines-09-01473-s001.zip › biomedicines-1395046-supplementary.pdf]
